# Simultaneous analyses of N-linked and O-linked glycans of ovarian cancer cells using solid-phase chemoenzymatic method

**DOI:** 10.1186/s12014-017-9137-1

**Published:** 2017-01-13

**Authors:** Shuang Yang, Naseruddin Höti, Weiming Yang, Yang Liu, Lijun Chen, Shuwei Li, Hui Zhang

**Affiliations:** 1 Department of Pathology, Johns Hopkins Medicine, Smith Bldg 4013, 400 N. Broadway, Baltimore, MD 21287 USA; 2 Institute for Bioscience and Biotechnology Research, University of Maryland College Park, Rockville, MD 20850 USA

**Keywords:** Chemoenzymatic, Glycoprotein, Glycomics, Solid phase

## Abstract

**Background:**

Glycans play critical roles in a number of biological activities. Two common types of glycans, N-linked and O-linked, have been extensively analyzed in the last decades. N-glycans are typically released from glycoproteins by enzymes, while O-glycans are released from glycoproteins by chemical methods. It is important to identify and quantify both N- and O-linked glycans of glycoproteins to determine the changes of glycans.

**Methods:**

The effort has been dedicated to study glycans from ovarian cancer cells treated with O-linked glycosylation inhibitor qualitatively and quantitatively. We used a solid-phase chemoenzymatic approach to systematically identify and quantify N-glycans and O-glycans in the ovarian cancer cells. It consists of three steps: (1) immobilization of proteins from cells and derivatization of glycans to protect sialic acids; (2) release of N-glycans by PNGase F and quantification of N-glycans by isobaric tags; (3) release and quantification of O-glycans by β-elimination in the presence of 1-phenyl-3-methyl-5-pyrazolone (PMP).

**Results:**

We used ovarian cancer cell lines to study effect of O-linked glycosylation inhibitor on protein glycosylation. Results suggested that the inhibition of O-linked glycosylation reduced the levels of O-glycans. Interestingly, it appeared to increase N-glycan level in a lower dose of the O-linked glycosylation inhibitor. The sequential release and analyses of N-linked and O-linked glycans using chemoenzymatic approach are a platform for studying N-glycans and O-glycans in complex biological samples.

**Conclusion:**

The solid-phase chemoenzymatic method was used to analyze both N-linked and O-linked glycans sequentially released from the ovarian cancer cells. The biological studies on O-linked glycosylation inhibition indicate the effects of O-glycosylation inhibition to glycan changes in both O-linked and N-linked glycan expression.

**Electronic supplementary material:**

The online version of this article (doi:10.1186/s12014-017-9137-1) contains supplementary material, which is available to authorized users.

## Background

Glycosylation is one of the most abundant and diverse protein modifications. It plays essential roles in the biological and physiological functions of a living organism [1]. Aberrant glycosylation is associated with different diseases, e.g. prostate cancer [2], ovarian cancer [3, 4], rheumatoid arthritis [5], diabetes [6], and cardiac diseases [7, 8]. Studies reveal that cancer cells often display their glycans at different levels of structures as compared to those observed on normal cells [9]. Glycosylation can thus be harnessed for defining cancer malignancy and disease progression [10, 11]. The abnormal glycosylation may contribute to cancer metastasis [12, 13]. Therefore, it is important to characterize protein glycosylation in biological and clinical specimens.

The N-linked and O-linked glycans are two most commonly studied glycoforms in protein glycosylation. The N-glycan has common core structure (GlcNAc_2_Man_3_) that conjugates to the asparagine (Asn or N) residues in the consensus peptide motif of Asn-X-Ser/Thr [where X is any amino acid except proline (Pro)]; The O-glycan conjugates to serine (Ser) or threonine (Thr) without a consensus amino-acid motif. The structure of glycans is complex due to its non-template biosynthesis pathway. The complexity is predominantly due to its variable monosaccharides, branches, linkages, and isomers.

It is preferable to analyze both N-glycans and O-glycans from glycoproteins; technology development to achieve this goal has been the focus for glycomics [14–20]. Release of these glycans from glycoproteins can be fulfilled by enzymes or chemical reactions. PNGase F (peptide: N-glycosidase F) releases all N-glycans except for glycans with core-α(1,3)-fucose that are found only in slime molds, plants, insects, and parasites plant and insect [21], whereas PNGase A (peptide-N4-(*N*-acetyl-β-glucosaminyl)asparagine amidase) releases these N-glycans from glycopeptides including core-α(1,3)-fucose and all N-glycans released by PNGase F [22]. However, no universal O-glycosidase has been developed for the removal of all O-glycans except for core 1 (Gal-GalNAc) or core 3 (GlcNAc-GalNAc). The removal of O-glycans is usually performed through alkali treatment using β-elimination [23, 24] or hydrazinolysis [25, 26]. Chemical release is cost-effective and can be ubiquitously applied to release different types of glycans. Hydrazine hydrolysis releases both O-glycans (60 °C) and N-glycans (95 °C) [26, 27]. However, even at a relatively lower temperature for O-glycans release (60 °C), it can still result in N-glycan release. The recently reported oxidative strategy releases all types of glycans including N-glycans and O-glycans without specificity [28]. It has been reported that O-glycans can be specifically released at a mild β-elimination such as ammonia [29]; however, others showed that ammonia (26–28%) alone could also release both N-glycans and O-glycans [14]. Additional consideration with glycans released by the chemical methods is the sequential degradation of reducing-end monosaccharide units by consecutive β-elimination, also known as “peeling” [30, 31]. The peeling of the alditols on the reducing end is showed to be prevented by release of O-glycans in a mild medium in the presence of reagents for alditol capping [32]. Several chemical compounds have been exploited for the capping of O-glycan alditol after β-elimination. Among them, pyrazolone derivatives have been used for capping the alditol and enhancing hydrophobicity of glycans for LC–ESI–MS [33, 34].

An integrated platform has been sought for the comprehensive profiling of glycans [16–20, 35]. Numerous N-glycan studies have shown that native sialic acid residues are fragile and may be easily lost during sample preparation and ionization in MALDI-MS [14, 36–38]. Stabilization by chemical methods such as amidation [37], methyl esterification [39], permethylation [18, 19, 40], and perbenzolylation [41] has been developed for analysis of sialylated glycans. For example, glycoproteins are systematically analyzed by immobilizing on polymer membranes for sequential release of N-glycans and O-glycans [35]. Structural analysis can be achieved via sialidases or exoglycosidases in coupling with porous graphitized liquid chromatography-mass spectrometry [18]. Mass spectrometric screening strategy is developed for characterizing glycan component of both glycosphingolipids and glycoproteins from a single sample [20]. These methods have been widely used for analysis of glycans in biological specimens, such as ovarian cancers from serum and cell lines [4, 42–44].

It has been successfully demonstrated that sialic acid residues can be effectively stabilized using an in-solution amidation [37, 45]. Permethylation of the released glycans can protect sialic acids for both N- and O-glycans [46, 47]. Yet, the decomposition of *O*-acetyl groups may occur under the harsh conditions used for permethylation [45]. Besides, the permethylated glycans may lose their reactivity on the reducing-ends, consequently preventing their further use for fluorophore, chromophore, or isobaric tag labeling [48]. To this end, we recently developed a solid-phase chemoenzymatic platform termed as glycoprotein immobilization for glycan extraction (GIG) by conjugating glycoproteins on solid phase, protecting sialic acids, and sequentially releasing N- and O-linked glycans for MS analyses [14, 49, 50].

Glycoprofiling on ovarian cancer serum found that unique N-glycans were present in cancer patient [4]. Profiling of N-glycans by a nanoLC mass spectrometric method observed up-regulation of the fucosylated N-glycans in healthy controls [51]. Recent works discovered the glycosylation changes in ovarian cancers were influenced by aberrant regulation of gene expression. The characteristic glycan features that were unique to the ovarian cancer membrane proteins have been identified, including “bi-secting *N*-acetyl-glucosamine” and “*N*,*N*′-diacetyl-lactosamine” type N-glycans [42]. These glycosylation changes in ovarian cancer may contribute to disease pathogenesis [44]. Therefore, inhibition of protein glycosylation may be useful for ovarian cancer treatment. In this study, we applied the quantitative glycomics to the analyses of both N- and O-linked glycans in ovarian cancer cells in the presence and absence of inhibitor for O-linked glycosylation. The glycosylation changes on both N-glycans and O-glycans are described.

## Experimental section

### Reagents and sample preparation

All chemicals were purchased from Sigma-Aldrich (St. Louis, MO) unless specified otherwise. Aminolink resin, spin columns (snap cap), and Zeba spin desalting columns were purchased from Life Technologies (Grand Island, NY). Alltech Extract-Clean Carbograph columns, analytical column [NanoViper, 75 μm (ID), 150 mm, 2 µm particle size], water, methanol, and acetonitrile (ACN) (HPLC grade) were purchased from Fisher Sci. (Waltham, MA). NaCl solution (5 M) was ordered from ChemCruz Biochemicals (Santa Cruz, CA). Chloroform was purchased from J.T. Baker (VWR, Radnor, PA). Cell lysis buffer consists of 1× PBS, 1% NP-40, 0.5% sodium deoxycholate (C_24_H_39_NaO_4_), 0.1% SDS, 2 mM EDTA, and 50 mM NaF. Micro-centrifuge tubes (1–2 mL) were purchased from Denville Scientific Inc. (Holliston, MA). Sep-Pak C18 1 cc Vac Cartridges (50 mg sorbent per cartridge, 55–105 μm particle size) were purchased from Waters Corporation. Peptide-N-glycosidase F (PNGase F), denaturing buffer (10×), and GlycoBuffer (G7; 10×) were from New England Biolabs (Ipswich, MA).

### OVCAR-3 cell culture and treatment

OVCAR-3 cell line (ATCC^®^ HTB-161^TM^) was purchased from ATCC (American Type Culture Collection). Cell culture was proceed according to ATCC protocol. The culture medium consists of RPMI-1640 (Thermo Fisher), 0.01 mg/mL bovine insulin (Sigma), and 20% fetal bovine serum (Sigma). OVCAR-3 cells were suspended in a 15-cm cell culture dish (Thermo Fisher). O-GalNAc inhibitor (Benzyl-α-GalNAc or BAG; Sigma) was dissolved in DMSO (Dimethyl sulfoxide; Sigma) (100 mM). A final concentration of BAG (0, 0.2, 1, 2 mM) was added to OVCAR-3 for 24-h treatment. Cells were washed by 1× PBS three times before harvest in 1.5 mL microcentrifuge tube, followed by cell lysis in 500 μL of 1× binding buffer. Protein concentration was determined by BCA assay (Thermo Fisher). One mg protein was used for glycan analysis.

### Protein immobilization on solid phase and N-glycan release

Proteins were first extracted from cells using cell lysis buffer. Proteins (1 mg) were denatured at 100°C for 10 min in 100 μL solution consisting of 10 μL 10× denaturing buffer and 90 μL deionized (DI) water. After Aminolink resin was pre-conditioned by 1× binding buffer (500 μL; 3×) (pH 10; 100 mM sodium citrate and 50 mM sodium carbonate) [52], the denatured proteins were mixed with resin in a spin column by adding 350 μL DI water and 50 μL 10× binding buffer. The reaction proceeded up to 4 h with mixing at room temperature, followed by incubation for another 4 h after adding 25 μL of 1 M NaCNBH_3_. Next, resin was rinsed using 500 μL 1× PBS (3×) (Thermo Fisher). The conjugation continued for 4 h in 500 μL 1× PBS in the presence of 50 mM NaCNBH_3_. The active aldehyde sites on the resin were blocked using 500 μL 1× Tris–HCl (50 mM NaCNBH_3_). After washing the resin using 1 M NaCl and DI water (500 μL, 3×), the sialic acid residues were reacted with 1 M p-Toluidine (pT, Sigma) buffer via carbodiimide coupling (3 h). The pT buffer (465 μL) consisted of 400 μL of 1 M pT, 25 μL HCl (36–38%), and 40 μL EDC (N-(3-dimethylaminopropyl)-N′-ethylcarbodiimide). To remove chemical compounds such as pT and EDC, the resin was extensively washed with four solutions (500 μL) in a sequential order of 10% formic acid (3×), 10% acetonitrile in 0.1% TFA (trifluoroacetic acid) (3×), 1 M NaCl (3×), and DI (3×). N-glycans were then enzymatically released by 2 μL PNGase F (1000 units; 360 μL DI, 40 μL 10× GlycoBuffer; 37 °C, 3 h). The released N-glycans were purified by Carbograph as described in our previous protocol [36].

### Chemical release of O-glycans from solid phase

**Fig. 1:**
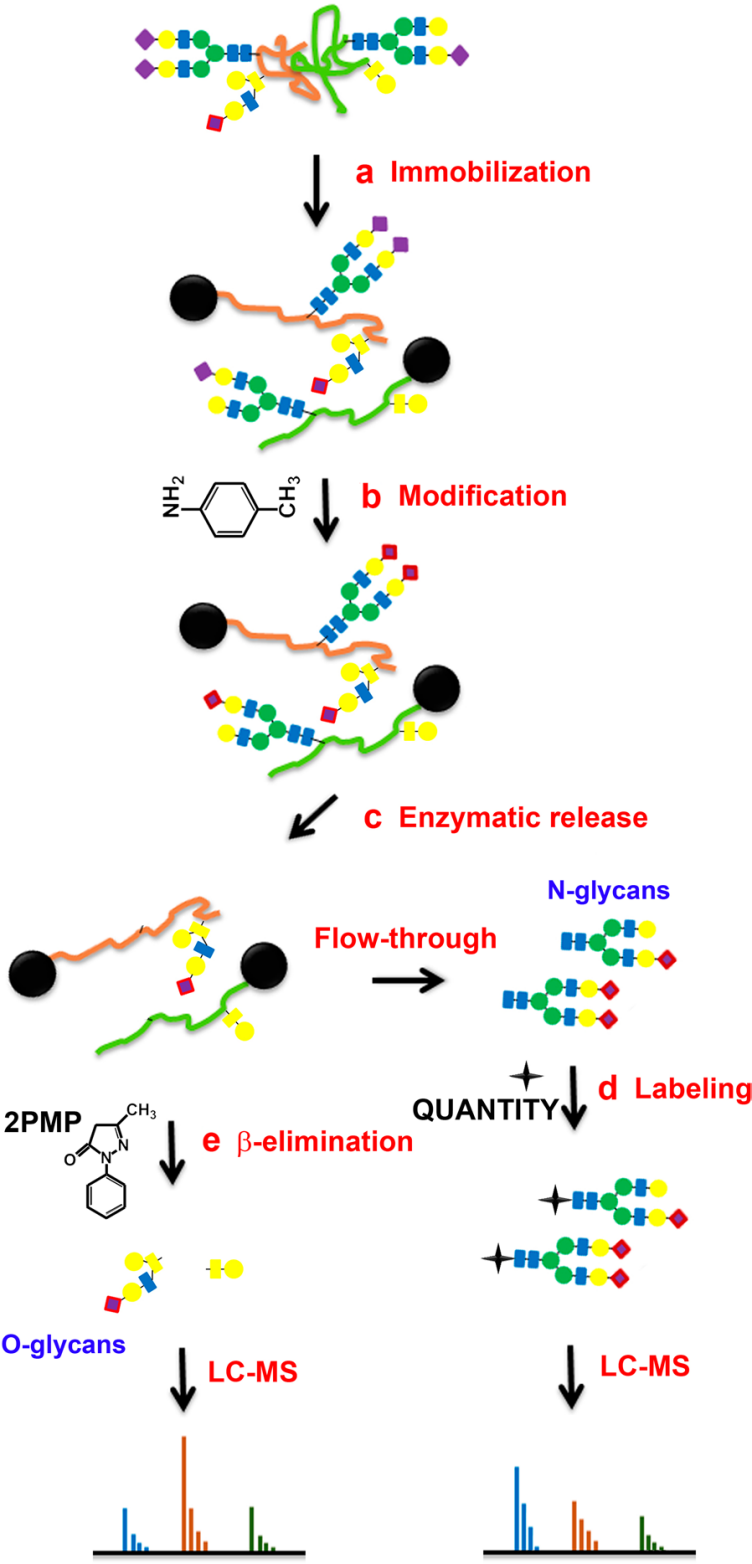
Schematic diagram of sequential releases and analyses of N-linked and O-linked glycans via chemoenzymatic method. **a** Immobilize glycoproteins on solid support. **b** Modify sialic acids; **c** release N-glycans using PNGase F; **d** label N-glycans by the isobaric tags such as QUANTITY via reductive amination; **e** release O-glycans by β-elimination. The released O-glycans are purified using C18 cartridge and N-glycans are purified using Carbograph SPE column

The resin was extensively washed with 1 M NaCl and DI (500 μL; 3×) after removal of N-glycans. Water was removed from the spin column by centrifuge (2000×*g*; 30 s) and the resin was transferred to a 2-mL micro-centrifuge tube. Two-hundred microlitre ammonia (NH_4_OH; 26–28%) and 300 μL 500 mM PMP in methanol were mixed with resin, resulting in a lower concentration of ammonia (11.2%). The mixture was vortexed and reacted at 55 °C for 24–48 h (Fig. 1e). The samples were transferred back to the spin column to collect the supernatant. Resin was washed with DI water (300 μL; 3×) and all flow-through fractions were combined with the previously collected supernatant. After being dried under vacuum (Savant SpeedVac, Thermo Scientific), samples were re-suspended in 200 μL acidic water (1% acetic acid) and 400 μL chloroform. The free PMP was completely mixed in chloroform while the labeled O-glycans were dissolved in 1% acetic acid. The excess PMP in chloroform was removed from the aqueous layer (water), and the extraction was repeated three more times (400 μL chloroform). The aqueous layer was dried under vacuum and re-dissolved in 1 mL of water (HPLC). The labeled O-glycans were purified using an SPE C18 cartridge, which was preconditioned with 1 mL 100% acetonitrile (2×) and 1 mL water (3×). The C18-SPE-loaded samples were rinsed with 1 mL water (5×) and eluted with 200 μL 50% acetonitrile (repeated once). The purified O-glycans were placed in a glass insert and dried under vacuum prior to LC–MS/MS analysis.

### Mass spectrometry and data analysis

MALDI (matrix-assisted laser desorption/ionization) was performed using Shimadzu Resonance Maxima QIT-ToF. Laser energy was 140–160; 1 μL of DHB (2,5-dihydroxybenzoic acid)-DMA (dimethylaniline) was mixed with 1 μL of glycans. The modified glycans were cleaned by a C18-SPE trap column (Thermo Scientific; Dionex nanoViper Fingertight Fitting). Sample (12 μL) was injected to the trap column (C18) by the loading pump at a flow rate of 5 μL/min. The nano-flow pump (Thermo Scientific; Dinoex UltiMate 3000) was set at a flow rate of 0.25 μL/min; the LC gradient was set from 4% (acetonitrile, 0.1% TFA) to 50% within 70 min using an analytical column (Fisher Scientific; Thermo Scientific Acclaim PepMap 100 C18). The full scan MS1 mass range was from 400 to 1800 Da (m/z) using positive mode (Thermo Scientific; Orbitrap Velos; collision-induced dissociation: 30%). The MS2 parameters were as follows: collision energy 29%, isolation width 2.0, m/z, activation time 0.2 ms, and HCD (high-energy collision dissociation). Dynamic exclusion included repeat count 2, repeat duration 25 s, exclusion list size 500, and exclusion duration 5 s. Glycan spectra were analyzed using Thermo Xcalibur Qual Browser. Glycan composition was determined by (1) precursor matching and further confirmed by MS2 fragments (Additional file 1: Figure S1, 37 MS/MS); and (2) database matching using CFG (http://www.functionalglycomics.org), GlycomeDB (http://www.glycome-db.org) and Glycosciences (http://www.glycosciences.de/database/index.php) for those low abundance glycans. Glycans without MS/MS were given by their composition (N: HexNAc; H: Hexose; F: Fucose; S: NeuAc). The figures depicting the glycan structures were plotted using Glycoworkbench 2.1 software [53].

## Results and discussion

**Fig. 2:**
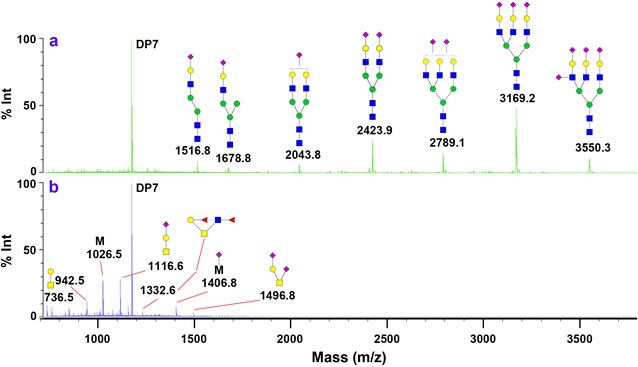
Chemoenzymatic sequential releases of N-glycans and O-glycans from bovine serum-derived fetuin using GIG. **a** N-glycans were released by PNGase F on solid-phase; **b** O-glycans were released after N-glycans were released by mild β-elimination in 0.5 M PMP (1-phenyl-3-methyl-5-pyrazolone). The MS spectra was generated by MALDI

**Fig. 3:**
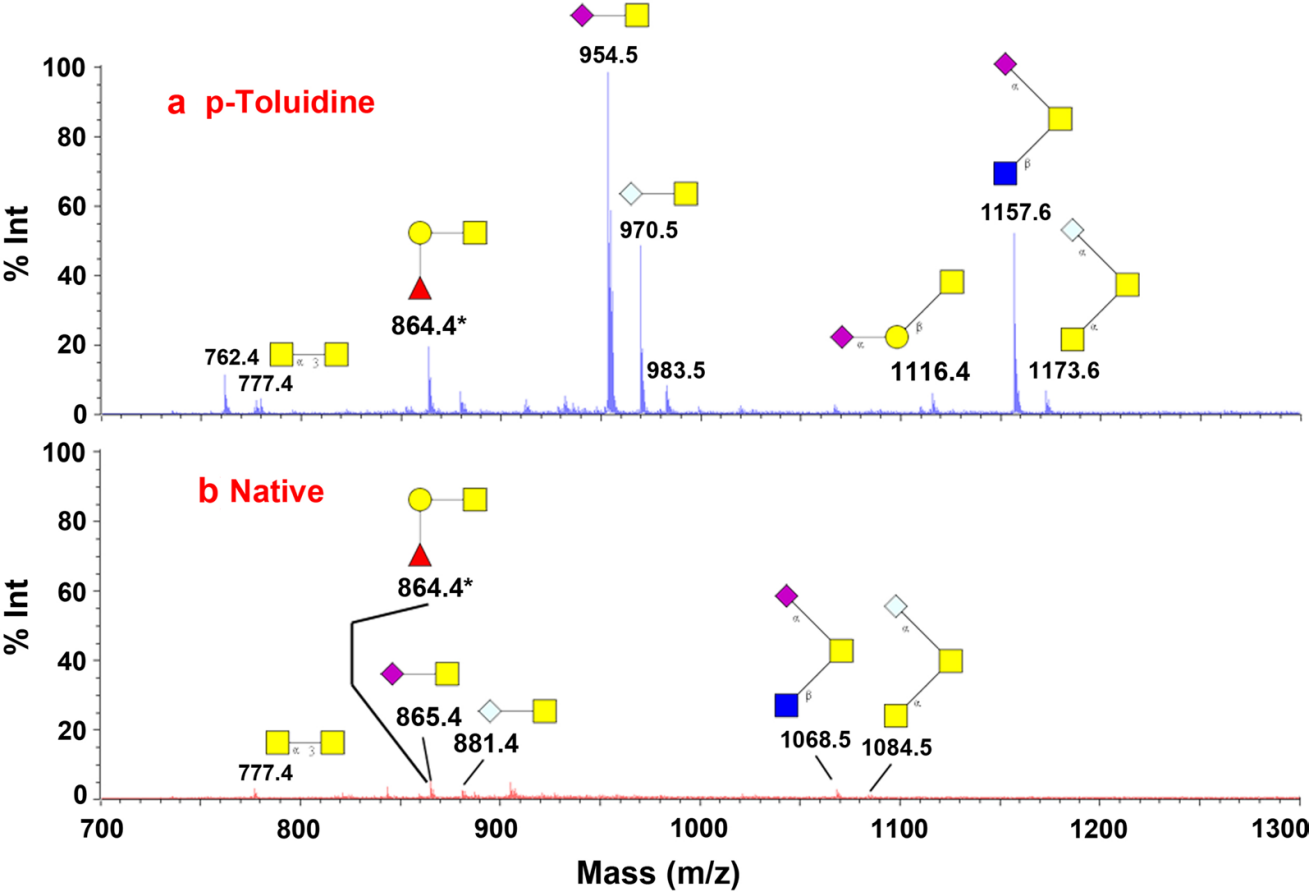
Sialylated O-glycans of mucin from bovine submaxillary glands (MBS) by MALDI-MS. **a** The sialic acids that were stabilized by carbodiimide coupling have a significantly increased MS signal; **b** the sialic acids without modification have low intensity in MALDI-MS. An internal standard (Neurotensin, 20 μM/1 μL) was spiked in the sample. The sialic acid modified glycans have one sodium adduct [Na]^+^, while native glycans have an extra sodium adduct per sialic acid

GIG consists of three steps: (1) the denatured proteins are conjugated on a solid support (amine-reactive resin (aldehyde)) via reductive amination (Fig. 1a); the immobilized proteins are modified via carbodiimide coupling on the solid support for stabilization of the sialic acids (Fig. 1b); (2) N-glycans are released by PNGase F treatment (Fig. 1c) and labeled with isobaric tags (QUANTITY) for relative quantification [49] (Fig. 1d); (3) O-glycans are released from the solid support via β-elimination using ammonia in the presence of PMP (Fig. 1e). The labeled N-glycans and O-glycans are identified and quantified by LC–MS/MS.

### Sequential release of N-glycans and O-glycans

To determine the performance of sequential release of N-and O-glycans from solid support, fetuin from bovine serum was conjugated on GIG resin to release glycans using PNGase F and ammonia. The first experiment was to determine the efficiency of N-glycan release by PNGase F, and then O-glycan release by β-elimination on the same sample. As shown in Fig. 2a, N-glycans are released directly from bovine fetuin conjugated on solid support by PNGase F digestion. The five major sialylated N-glycans are shown in Fig. 2a. Use the same specimen after N-glycan release, O-glycans were cleaved while their reducing-end alditols are protected by PMP [54]. The highly abundant O-glycans in fetuin include sialylated O-GalNAc, i.e. NHS (DP7 was spiked as an internal standard) (Fig. 2b), which is in agreement with the results from recent chromatographic analysis [55]. These results indicate that N-glycans and O-glycans can be cleaved from their amino acid on the solid support.

### Protection of sialylated O-glycans

The sialic acids are fragile and preferentially lost during sample preparation and ionization in MS. Sialic acid is negatively charged and hydrophilic, thus its identification is ineffective in the positive ionization mode for MS. The negative ionization mode is commonly used and has been well developed for the analysis of intact sialic acids [56, 57]. Modification of sialic acid provides several advantages: (1) stabilization of sialic acids, (2) neutralization of negative charge, and (3) enhanced hydrophobicity. Similar to modification on N-glycans [58], the sialic acid residues of O-glycans are simultaneously protected via carbodiimide coupling (Fig. 1b).

To demonstrate sialic acid modification on O-glycan analysis using GIG, mucin from bovine submaxillary glands (MSB) was immobilized on resin using the detailed protocol described in our previous studies [14, 36]. MALDI-MS profiling was used to compare the relative abundance of the sialylated O-glycans that are chemically released from MSB with sialic acid modification (Fig. 3a) and without modification (Fig. 3b). To estimate the signal between (a) and (b), an internal peptide standard (Neurotensin, Sigma) was spiked in the MALDI matrix (20 μM/1 μL). The intensity of Neurotensin is approximately the same (1000 mV) in (a) and (b). As shown in Fig. 3a, four major sialylated O-glycans are identified after sialic acid modification, including NS, NG, N_2_S, and N_2_G, which are listed in order of descending relative abundance. This result is consistent with findings reported in the literature [31].

### Analyses of N- and O-glycans from ovarian cancer cells treated with O-glycosylation inhibitor

We then applied the sequential release and analyses of N- and O-linked glycans from OVCAR-3 cells treated with Benzyl-α-GalNAc (BAG) to inhibit α-GalNAc biosynthesis. Different concentrations of BAG (0 mM (control), 0.2, 1, and 2 mM) were used to treat OVCAR-3 cells for 24 h. Cells were harvested and proteins were extracted. After protein (1 mg for each sample) immobilization, N-glycans were first released by PNGase F, followed by Carbograph cleanup [59]. One tenth of the N-glycans was loaded onto MALDI-MS for comparing the glycan profile from OVCAR-3 cells treated with different concentrations of BAG. An internal standard (25 μM/1 μL DP7) was used to determine the abundance of N-glycans as indicated in the Additional file 2: Table S1 (MALDI-OV3-Nglycan). Several observations are evident from the MALDI-MS analysis of N-glycans: (1) Oligomannoses are highly abundant N-glycans in OVCAR-3 cells; (2) Among the abundant oligomannose glycans, Man6 is the most abundant compared to other oligomannose glycans; and (3) Most oligomannoses are upregulated in 0.2 mM BAG-treated cells. The MALDI-MS profile of BAG-treated cell lines indicated that oligomannoses are highly abundant N-linked glycans in OVCAR-3 cells and affected by treatments using different concentrations of BAG.

**Fig. 4:**
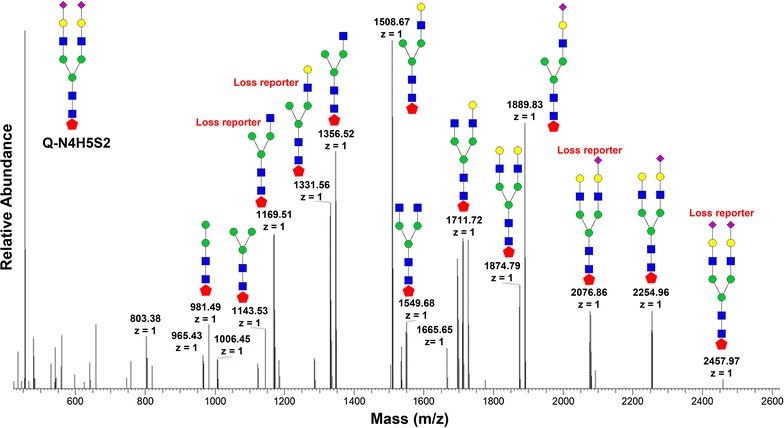
MS/MS fragmentation of QUANTITY-tagged N-glycans. The N4H5S2 was extracted from OVCAR-3 cells and labeled by QUANTITY. MS/MS was performed by Thermo Orbitrap Mass Spectrometer. When a reporter is lost, the mass is reduced by 176–178 with a “Loss reporter”

**Fig. 5:**
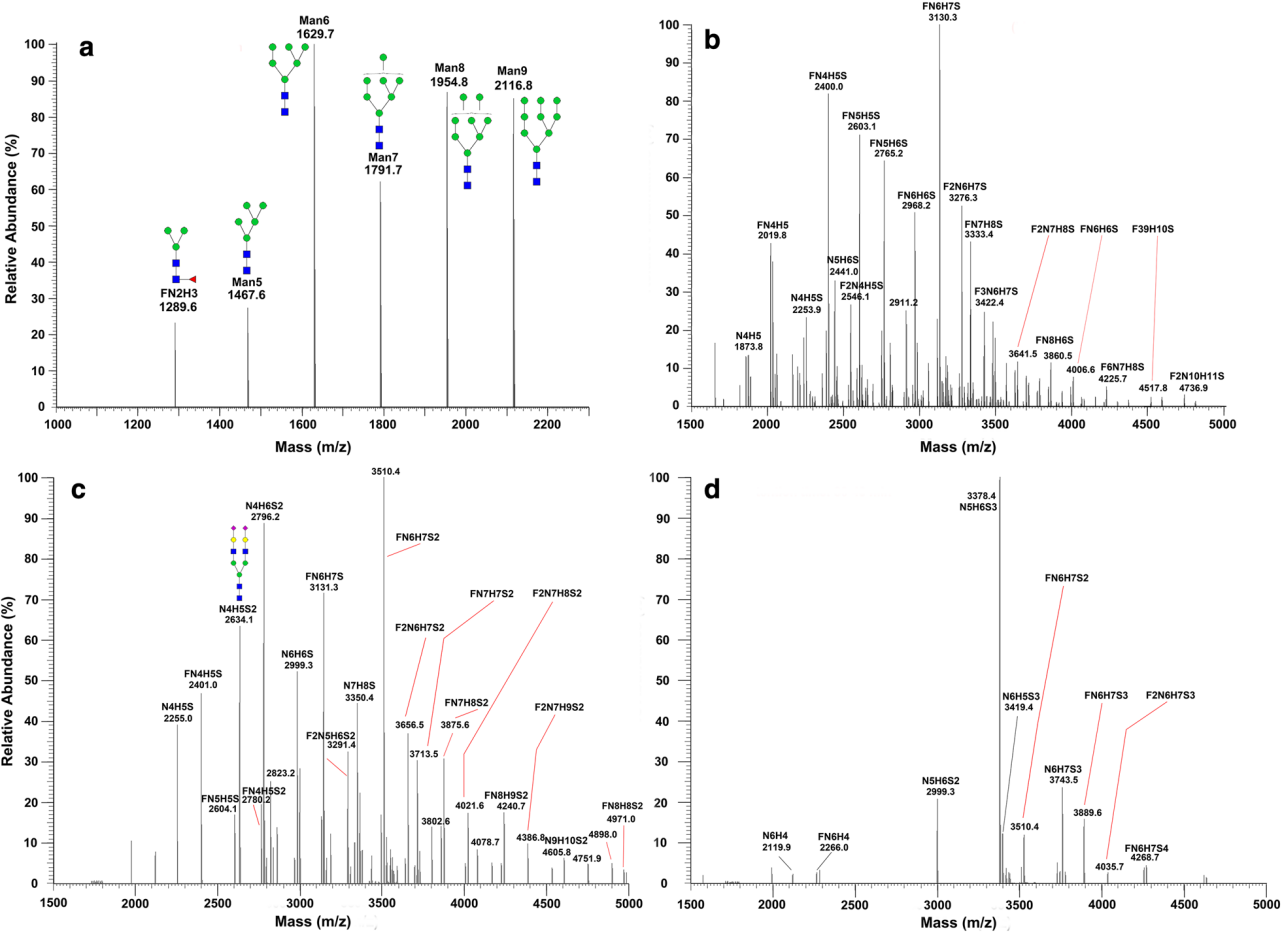
N-glycan profile of OVCAR-3 cells by LC–ESI–MS/MS. N-glycans were first released after sialic acid modification, and the released N-glycans were labeled using isobaric QUANTITY tags (Quaternary Amine Containing Isobaric Tag for Glycan). The labeled N-glycans were separated using a C18 analytical column (Thermo Scientific Acclaim PepMap, 15 cm). **a** Oligomannoses eluted from 0 to 10 min, **b** complex N-glycans eluted from 10 to 20 min, **c** Complex N-glycans eluted from 20 to 30 min, and **d** complex and sialylated N-glycans eluted from 30 to 40 min

To quantify N-glycans, the released N-glycans were also labeled with 4-plex isobaric tags (QUANTITY) for quantitative analysis by ESI–MS (Thermo; Orbitrap Velos Mass Spectrometer) [49]. Figure 4 shows the MS/MS fragmentation ions of N-glycans labeled with QUQNAITY, from which the cartoon structure is determined. A total of 137 N-glycans were identified and the high abundant N-glycans are highlighted in Fig. 5 and summarized in Additional file 2: Table S1 (LC-ESI-OV3-Nglycan). Among them, MS/MS spectra from the highest abundant glycans were generated (Additional file 1: Figure S1). After sialic acid labeling and reducing end tagging with QUANTITY, the hydrophobicity of N-glycans is significantly enhanced [50]. This allows the separation of the modified N-glycans on a C18 analytical column (15 cm in length) with the elution of oligomannoses first (Fig. 5a), followed by complex and highly sialylated N-glycans (Figs. 5b, c, d). Using a linear gradient from 4% ACN to 50% ACN over a 70 min period, the retention time is (a) 0–10 min for oligomannoses, (b) 10–20 min for complex glycans, (c) 20–30 min for complex glycans with high-branch structures, and (d) 30–40 min for complex sialylated glycans. In general, N-glycans were upregulated in the BAG-treated cells. Quantitative analysis by QUANTITY shows that 35 N-glycans were significantly upregulated by BAG treatment at a concentration of 1 mM (Additional file 3: Table S2).

The detail mechanism of N-glycan upregulation in BAG treated ovarian cancer cells is unclear. It has been indicated that the glycosylation of proteins in Golgi and in-transit glycoproteins could be affected by BAG [60]. Several polypeptide-*N*-acetyl-galactosaminyltransferases (ppGalNAcTs) are located throughout the Golgi, where N-glycans are synthesized. BAG inhibition could essentially affect many transcriptional factors that may regulate genes associated with N-glycan synthesis [61]. Therefore, the inhibition of O-GalNAc glycans might indirectly affect N-glycan biosynthesis [62].

BAG is a compound that acts as a competitive substrate for the synthesis of core 1, core 2, core 3, and core 4 O-GalNAc glycans in cells. It thus leads to a reduction in the synthesis of complex O-GalNAc glycans [29, 63]. The dominant O-glycans (26) are present in Table 1 (132 possible O-glycans were assigned using precursor matching as described in the Additional file 4: Table S3). Based on the change of O-GalNAc glycans under different BAG concentrations, the abundance of eight O-GalNAc glycans was reduced in BAG treated OVCAR-3 cells, including NS, N_2_H_2_, NHS, FN_2_H_2_, F_2_N_2_H_2_, N_3_H_3_, FN_3_H_2_S and N_2_H_2_S. However, few O-GalNAc glycans (e.g., N_3_HS) shows negligibly reduced or even no change by BAG, suggesting their biosynthesis being affected by other factors (the complete list is given in the Additional file 4: Table S3).

**Table 1:**
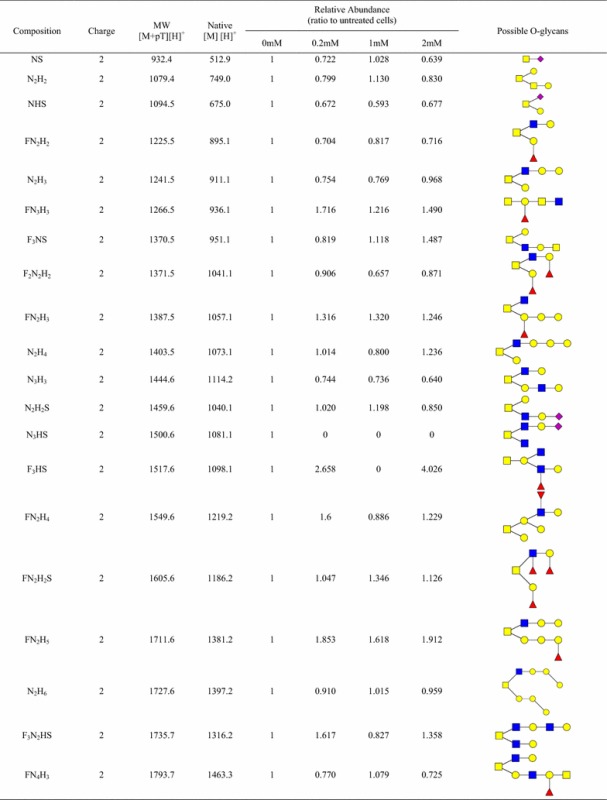

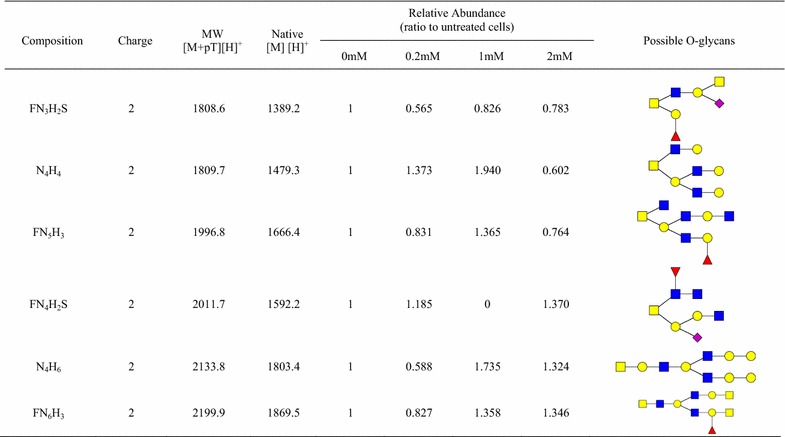
O-glycans identified from OVCAR-3 cells treated with the inhibitor Benzyl-α-GalNAc (BAG) using solid-phase chemoenzymatic method

BAG inhibitor was added to the cell medium for 24-h incubation before cell harvest. The concentration of BAG inhibitor is 0 (control), 0.2, 1, and 2 mM. *F* fucose, *N* HexNAc, *H* hexose, *S* Neu5Ac. (Standard deviation ≤10%) (The relative abundance is calculated by percentage of coverage from LC–MS/MS data)

Mucin-type O-glycans are critically regulated in cancers. For example, when CA125, an ovarian cancer marker, purified from the spent media of OVCAR-3 cells, O-glycomic analysis revealed that the sialylated O-glycans were highly abundant, containing NS, NHS and N_2_H_2_S; three dominant non-sialylated O-glycans were N_2_H_2_, N_3_H_2_, and N_3_H_3_ [64]. Our results indicate that the sialylated O-glycans in OVCAR-3 cells are effectively inhibited by BAG; however, non-sialylated O-glycans remain minimally regulated by inhibition of O-glycan biosynthesis. These observations are consistent with previous studies, indicating that BAG inhibition leads to a decrease of mucus secretion and a decreased intracellular amount of sialic acid [60, 63]. For example, BAG can impede the sialylation of O-glycosidic sugar chains on CD44, and the inhibition enhances experimental metastatic capacity in melanoma cells [65]. Subsequent studies have explored the possibility that the change of sialic acids in cells might be a consequence of the metabolic processing of BAG into Gal-BAG, which is a potent competitive inhibitor of the Gal-GalNAc-α2,3-sialyltransferase [62, 66]. Further inhibition of O-GalNAc glycosylation can be achieved by increasing the concentration of BAG (4–8 mM) and extending the treatment up to 72 h [61, 64, 67].

## Conclusion

A streamlined approach is used for the systematic identification and quantification of N-linked and O-linked glycans in the ovarian cancer cells. The performance of the platform is evaluated by the analysis of glycans in standard N- and O-linked glycoproteins. The stabilization of sialic acids by carbodiimide coupling to the solid support enhances the detection of sialylated glycans, which are not observed without sialic acid modification using in-solution β-elimination.

Inhibition of ovarian cancer cells by an O-GalNAc-targeted inhibitor appears to up-regulate N-glycans and down-regulate mucin-type O-glycans by two independent experiments using label-free glycomic analysis and isobaric labeled N-glycan analysis. To our knowledge, this is the first report to show the levels of N-glycans are regulated by O-linked glycosylation by O-GalNAc inhibitor. Even though the mechanism of this regulation is unclear, results indicate that a low concentration of O-GalNAc inhibitor might favor the biosynthesis of N-glycans in OVCAR-3 cells. The regulation of glycosylation biosynthesis by drugs should include considerations of their effects on both N-linked and O-linked glycans.

## Additional files

**Additional file 1: Figure S1.** MALDI-MS identification of Oligomannoses. The internal standard (1 μL, 25 μM DP7) is spiked in the matrix for semi-quantification. Note: Man5 = F0N2H5S0.

**Additional file 2: Table S2.** LC-ESI-MS quantification of OVCAR-3 cell lines released by PNGase F via solid-phase chemoenzymatic method. The released N-glycans are labeled with QUANTITY tags (4-plex). The labeled N-glycans are pooled for analysis by LC-ESI-MS. Oliogosaccharide and complex N-glycans are listed by their composition. (QU = QUANTITY; RT = retention time; Y = identified)

**Additional file 3: Table S3.** N-glycan (35) relative quantification after BAG treatment. Composition F = fucose; N = HexNAc; H = Hexose; S = Sialic acid; pT = p-Toluidine; Qu = Quantity. Fold change is calculated by intensity of N-glycan from BAG-treated cells versus that from non-treated OVCAR-3 cells.

**Additional file 4: Table S4.** Regulation of O-glycans by O-GalNAc inhibitors (BAG) on OVCAR-3 cells. N = HexNAc, H = Hexose, F = fucose, S = Sialic acid. The relative abundance is calculated by normalization using total intensity.

## Abbreviations

GIG: glycoprotein immobilization for glycan extraction
HPLC: high-performance liquid chromatography
ESI: electrospray ionization
MS: mass spectrometry
MALDI: matrix assisted laser desorption/ionization
QUANTITY: Quaternary Amine Containing Isobaric Tag for Glycan
DI: deionized water
