# Supplementary material for: Simultaneous analyses of N-linked and O-linked glycans of ovarian cancer cells using solid-phase chemoenzymatic method
Source: Clin Proteomics. 2017 Jan 13;14:3. doi: 10.1186/s12014-017-9137-1 (PMC5237303; doi:10.1186/s12014-017-9137-1)

**SUPPORTING INFORMATION FOR**

**Simultaneous analyses of N-linked and O-linked glycans of ovarian cancer cells using solid-phase chemoenzymatic method**

Shuang Yang^1*^, Naseruddin Höti^2^, Weiming Yang^3^, Yang Liu^4^, Lijun Chen^5^, Shuwei Li^6^, and Hui Zhang^7*^

^1^ Department of Pathology, Johns Hopkins Medicine, Baltimore, MD, USA, 21287

* Corresponding Author: Shuang Yang, Department of Pathology, Johns Hopkins Medicine, Smith Bldg 4013, 400 N. Broadway, Baltimore, MD, USA; Tel: (+1) 410-302-4681; Email: [jake.yang@gmail.com](mailto:jake.yang@gmail.com)

^2^ Department of Pathology, Johns Hopkins Medicine, Baltimore, MD, USA, 21287; Email: [nhoti1@jhmi.edu](mailto:nhoti1@jhmi.edu)

^3^ Department of Pathology, Johns Hopkins Medicine, Baltimore, MD, USA, 21287; Email: [wyang21@jhmi.edu](mailto:wyang21@jhmi.edu)

^4^ Department of Pathology, Johns Hopkins Medicine, Baltimore, MD, USA, 21287; Email: [yliu204@jhmi.edu](mailto:yliu204@jhmi.edu)

^5^ Department of Pathology, Johns Hopkins Medicine, Baltimore, MD, USA, 21287; Email: [lchen105@jhmi.edu](mailto:lchen105@jhmi.edu)

^6^ Institute for Bioscience and Biotechnology Research, University of Maryland College Park, Rockville, MD, USA, 20850; Email: [liw@ibbr.umd.edu](mailto:liw@ibbr.umd.edu)

^7^ Department of Pathology, Johns Hopkins Medicine, Baltimore, MD, USA, 21287; Email: [hzhang32@jhmi.edu](mailto:hzhang32@jhmi.edu)

**Abstract**

**Background:** Glycans play critical roles in a number of biological activities. Two common types of glycans, N-linked and O-linked, have been extensively analyzed in the last decades. N-glycans are typically released from glycoproteins by enzymes, while O-glycans are cleaved by β-elimination. It is important to identify and quantify N- and O-linked glycans to determine the changes of glycans in response to regulation of protein glycosylation.

**Methods:** The effort has been dedicated to study glycans from ovarian cancer cells treated with O-linked glycosylation inhibitor qualitatively and quantitatively. A solid-phase chemoenzymatic approach to the systematic identification and quantification of N-glycans and O-glycans in the ovarian cancer cells is described. It consists of three steps: (1) immobilization of proteins from cells and derivatization of glycans to protect sialic acids; (2) release of N-glycans by PNGase F and quantification of N-glycans by isobaric tags; (3) release and quantification of O-glycans by β-elimination in the presence of 1-phenyl-3-methyl-5-pyrazolone (PMP).

**Results:** We used ovarian cancer cell lines to study effect of O-linked glycosylation inhibitor on protein glycosylation. Results suggested that the inhibition of O-linked glycosylation reduced the levels of O-glycans. Interestingly, it appeared to increase N-glycan level in a lower dose of the O-GalNAc inhibitor. The sequential release and analyses of N-linked and O-linked glycans using chemoenzymatic approach are a platform for studying N-glycans and O-glycans in complex biological samples.

**Conclusion:** The solid-phase chemoenzymatic method was used to analyze both N-linked and O-linked glycans sequentially released from the ovarian cancer cells. The biological studies on O-GalNAc inhibition indicate the effects of O-glycosylation inhibition to glycan changes in both O-linked and N-linked glycan expression.

**Supporting Information Figure S1. MS/MS spectra of N-glycans from ovarian cancer cells.** N-glycans were released from solid-phase resin after sialic acid modification via p-Toluidine carbodiimide coupling. Each precursor was selected from mass spectrum of MALDI-MS by Shimadzu Axima Resonance. MS/MS was performed at collision energy 200. A total of 37 MS/MS was acquired from the most abundant N-glycans.


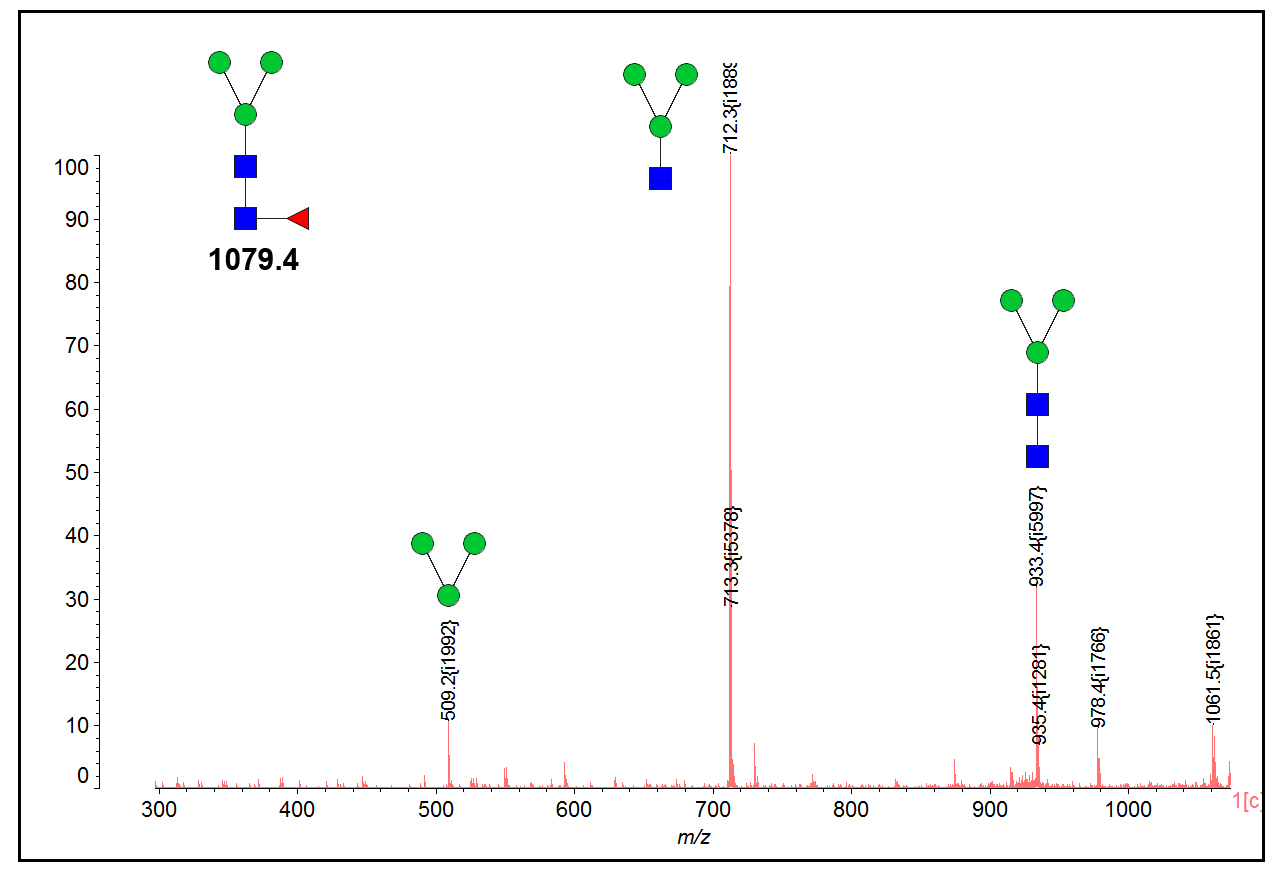

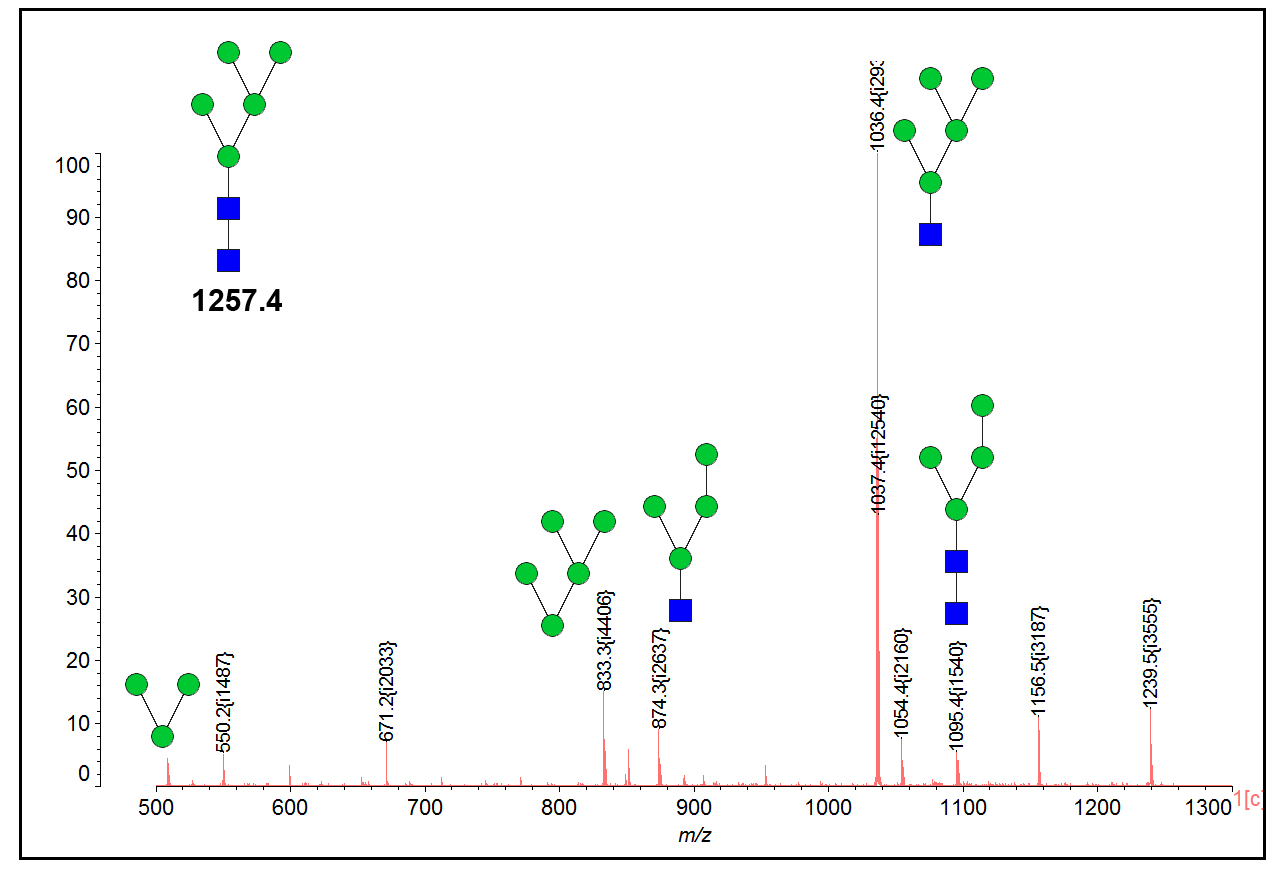

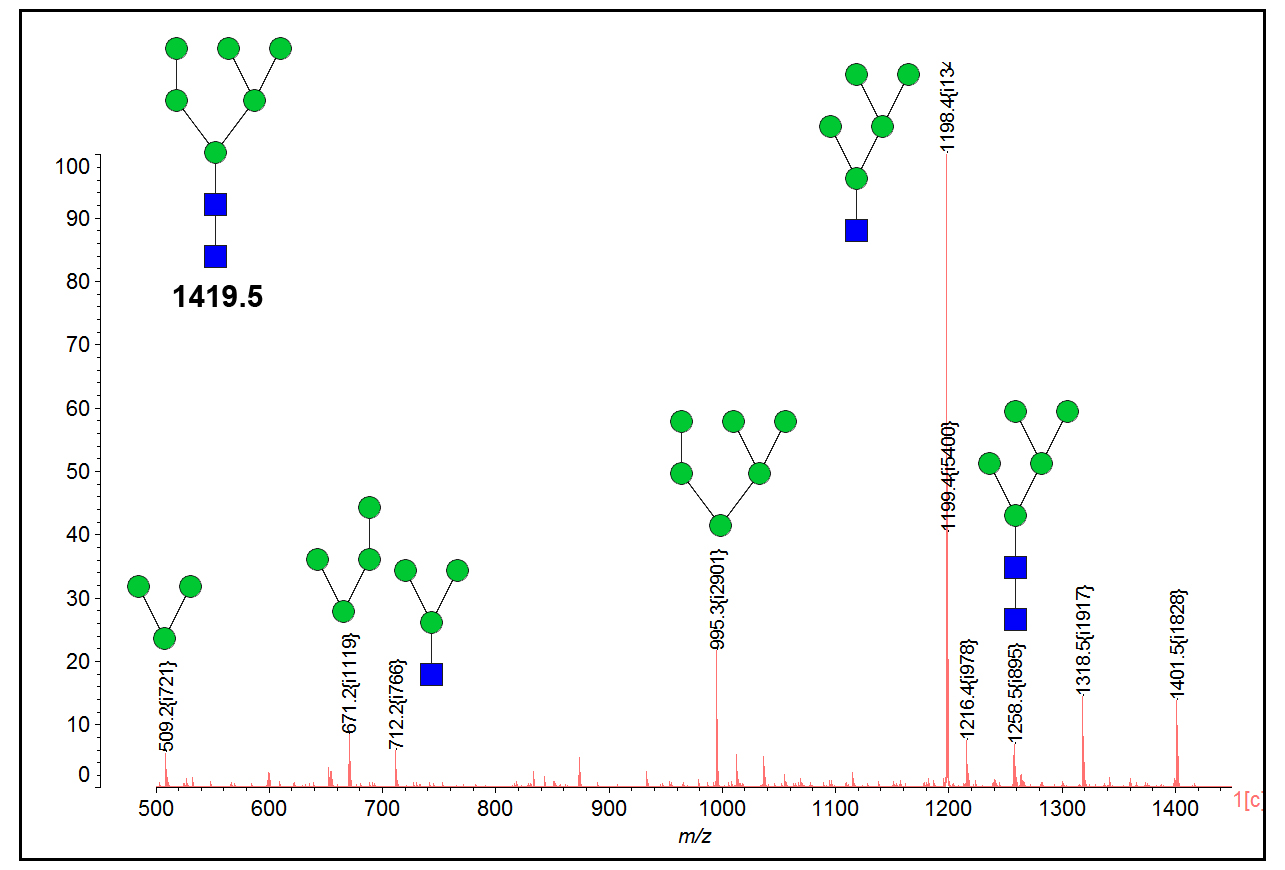

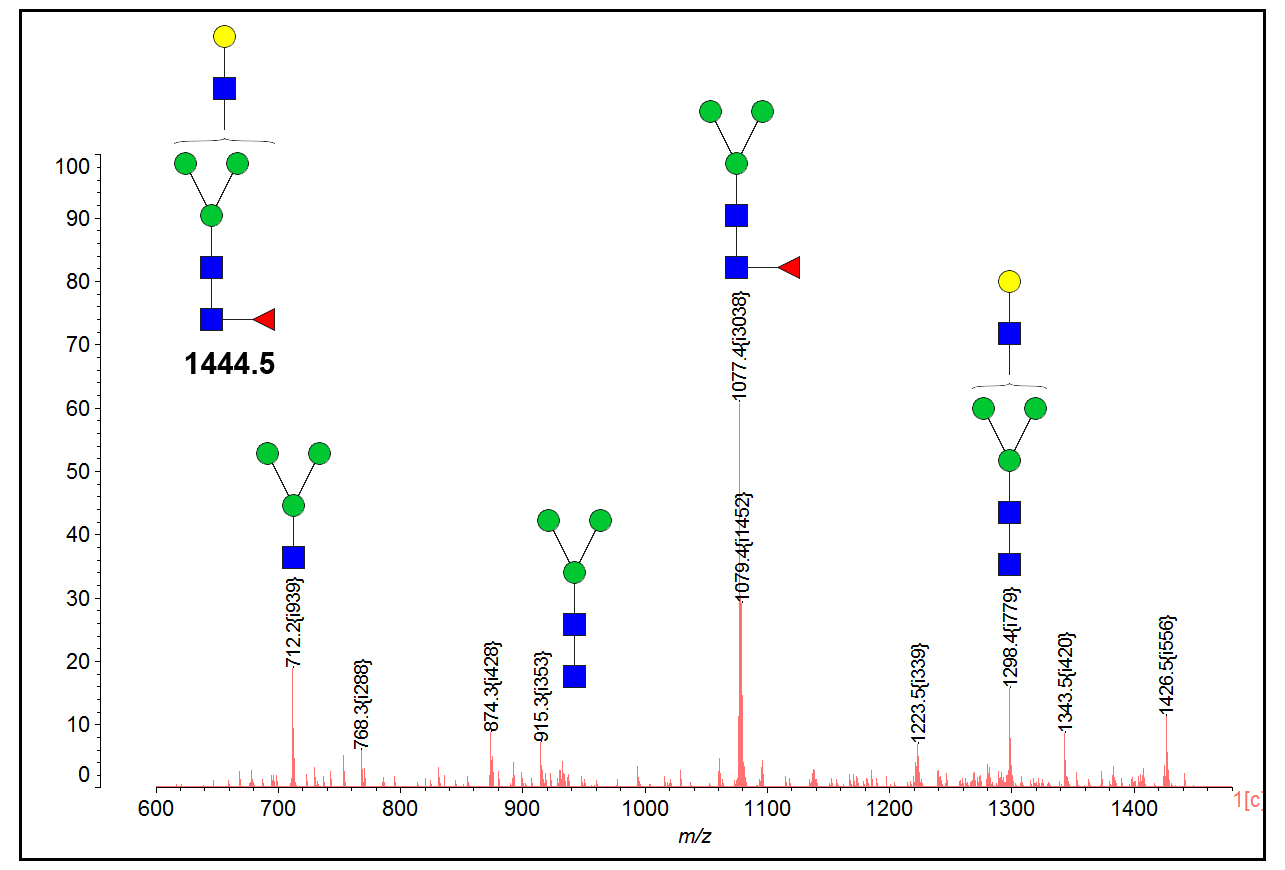

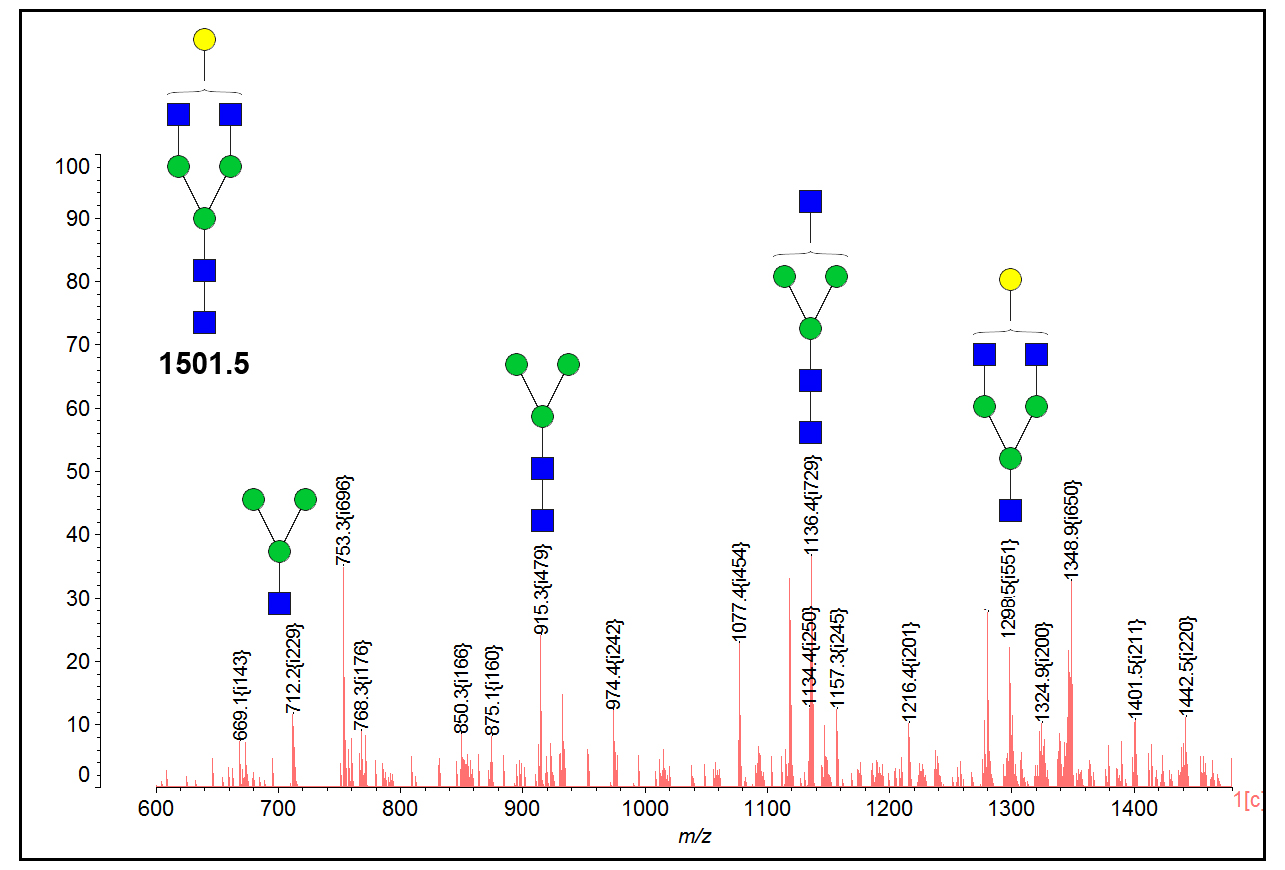

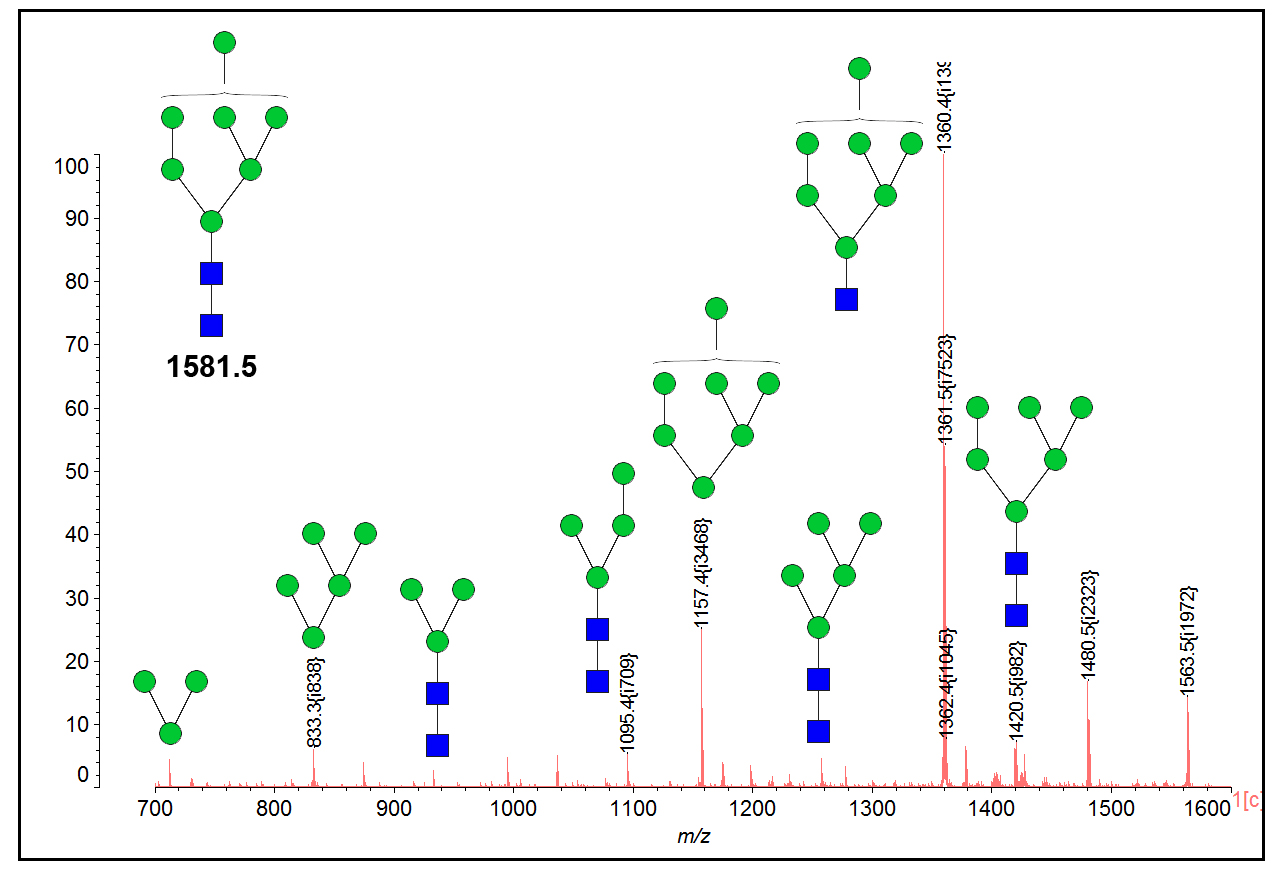

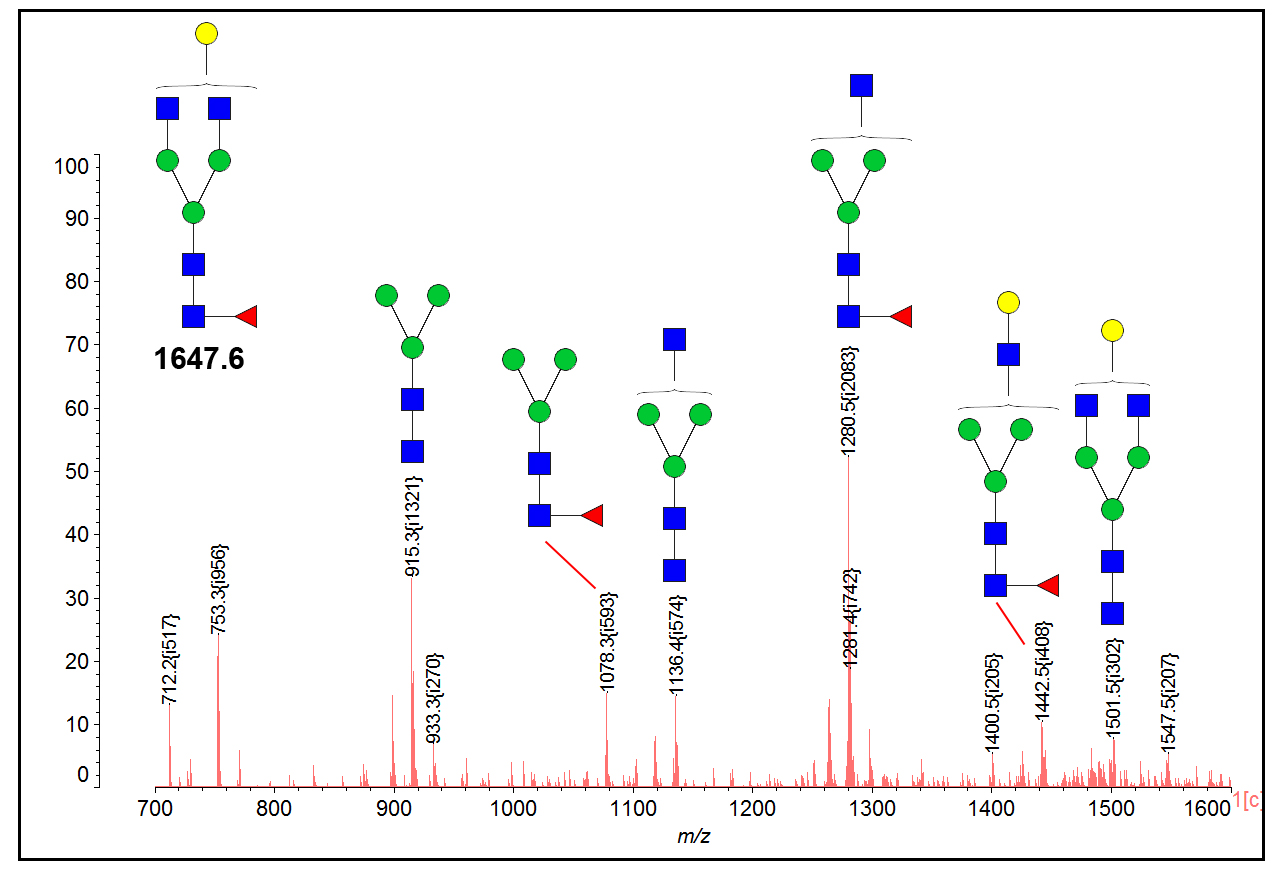

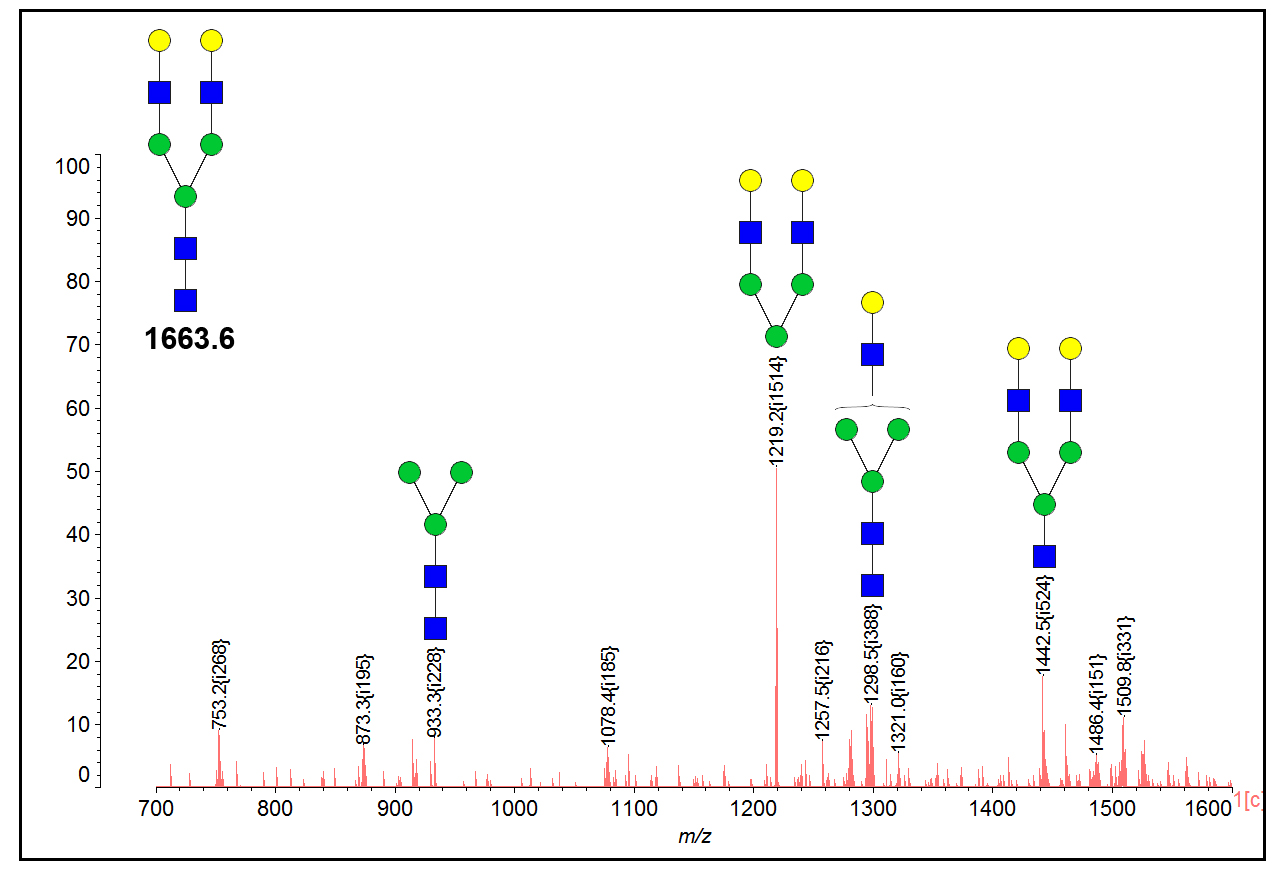

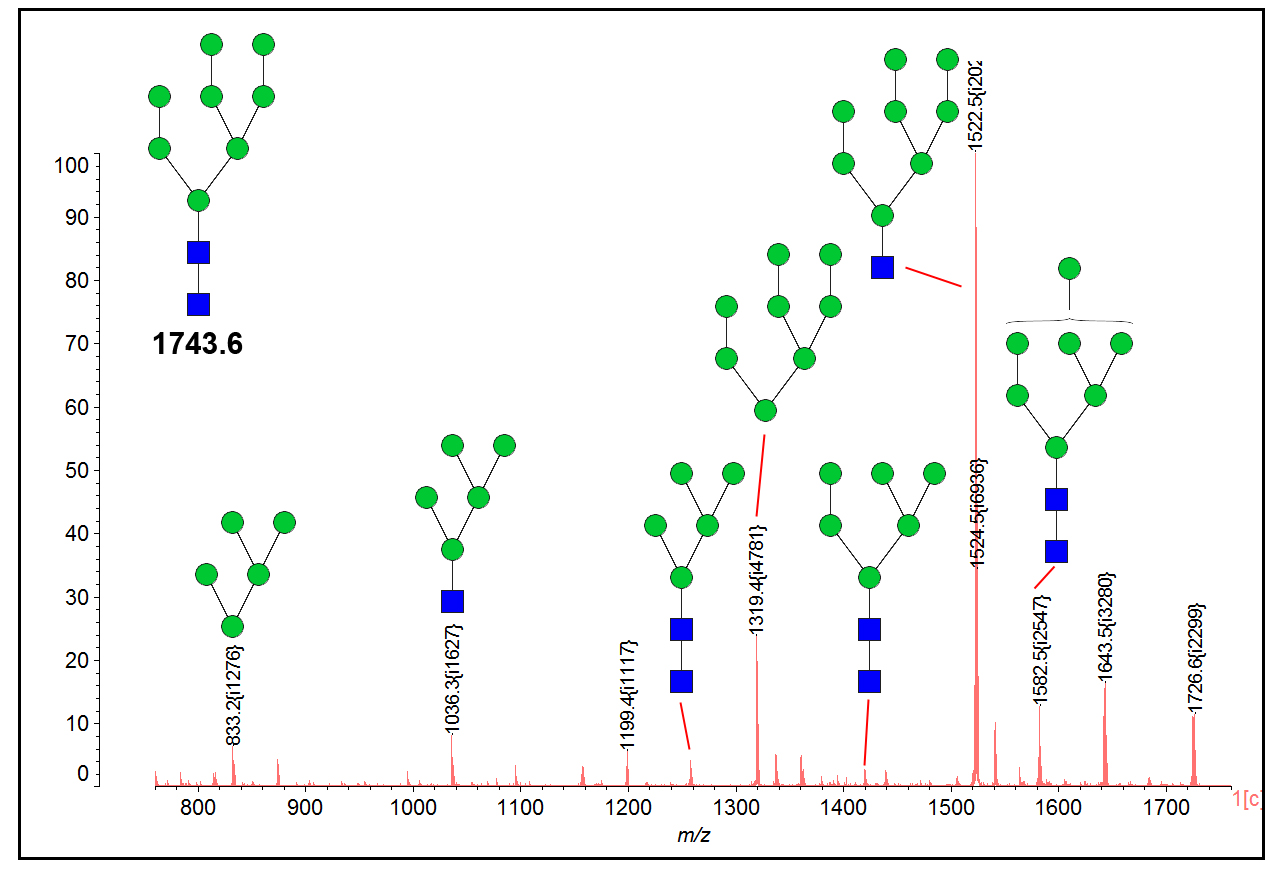

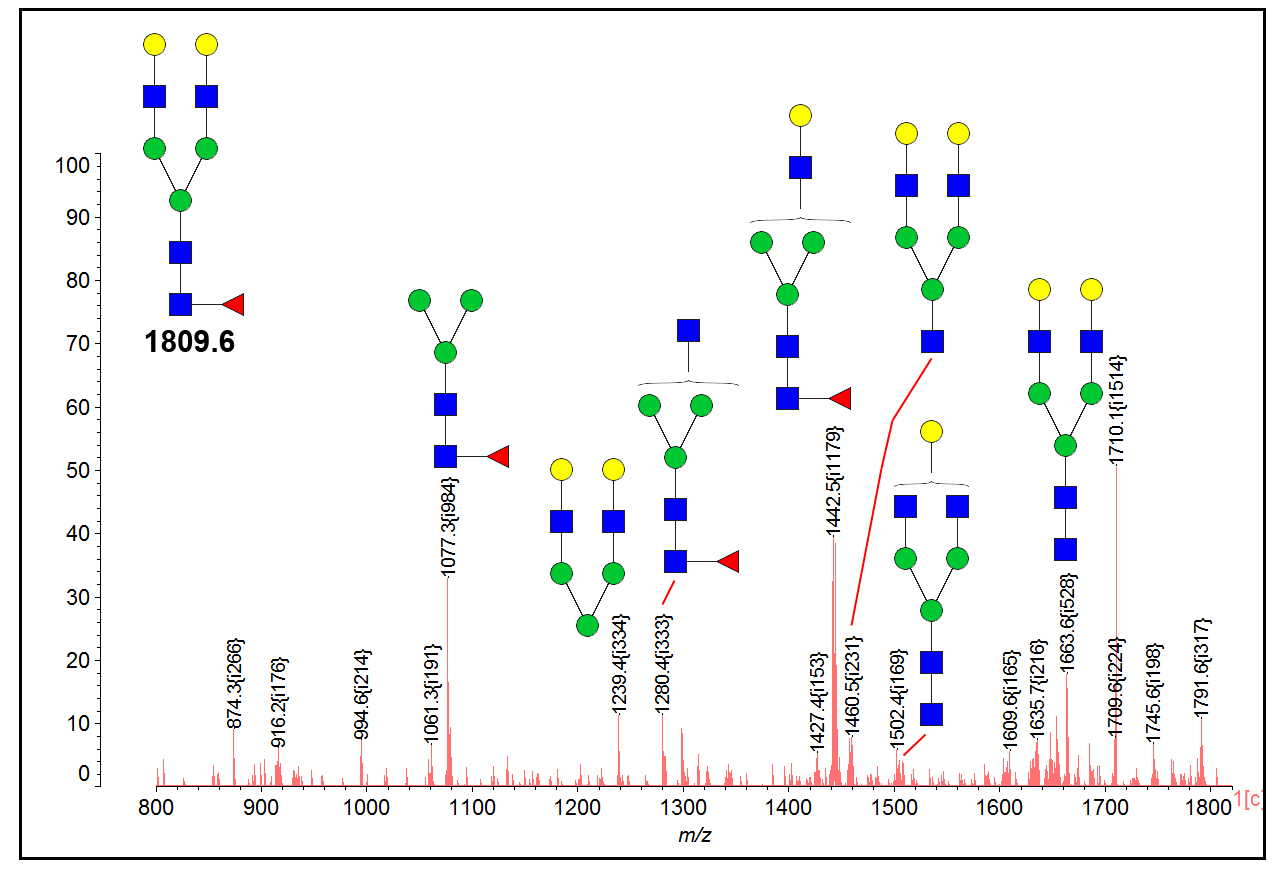

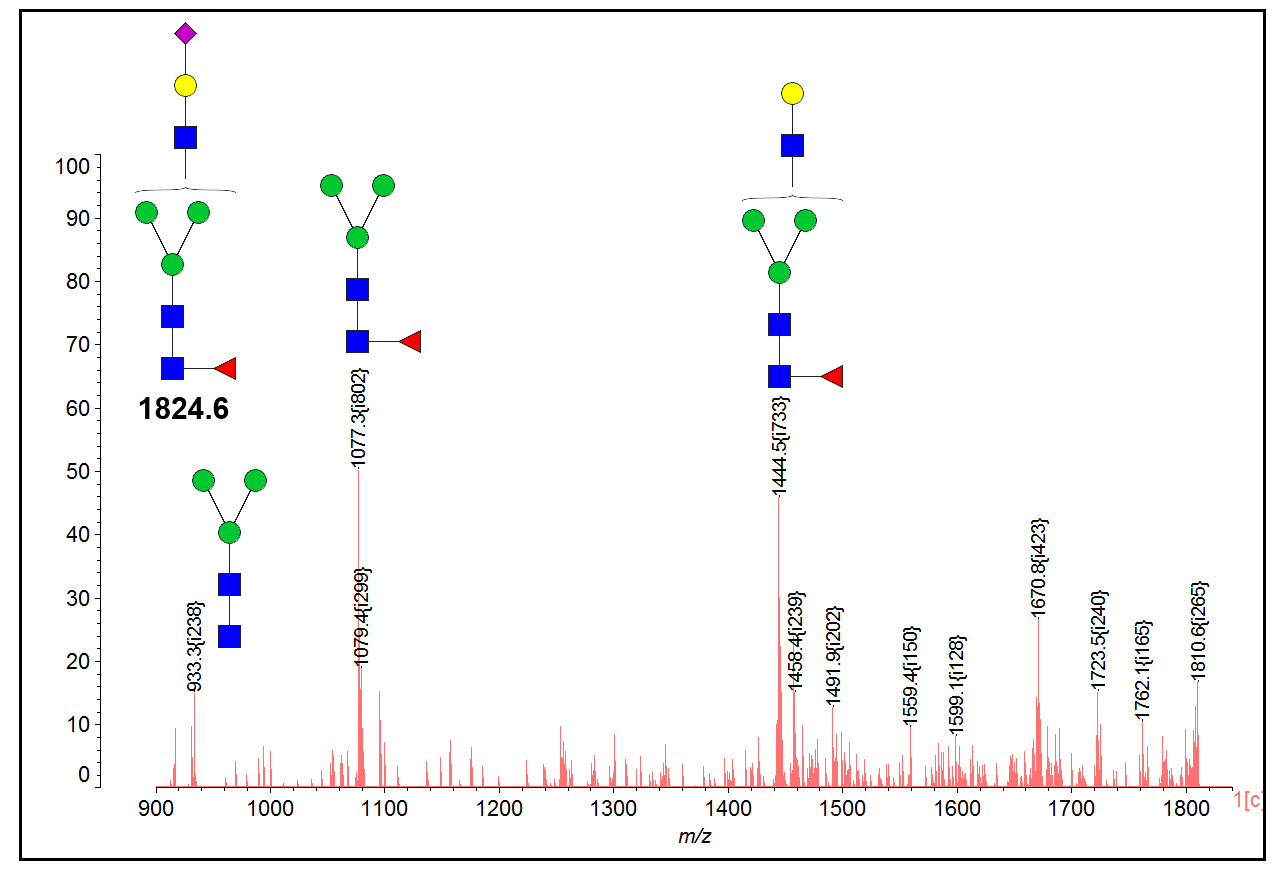

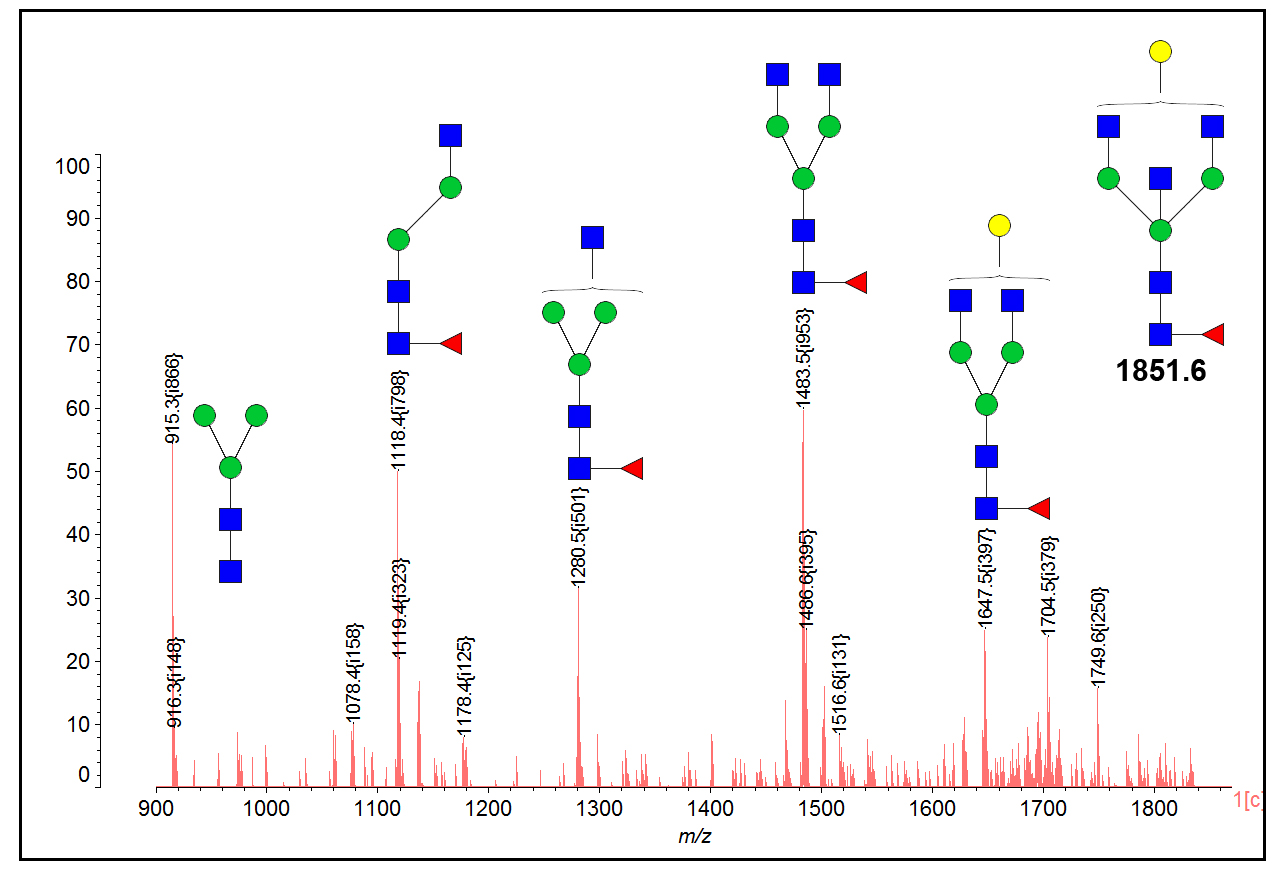

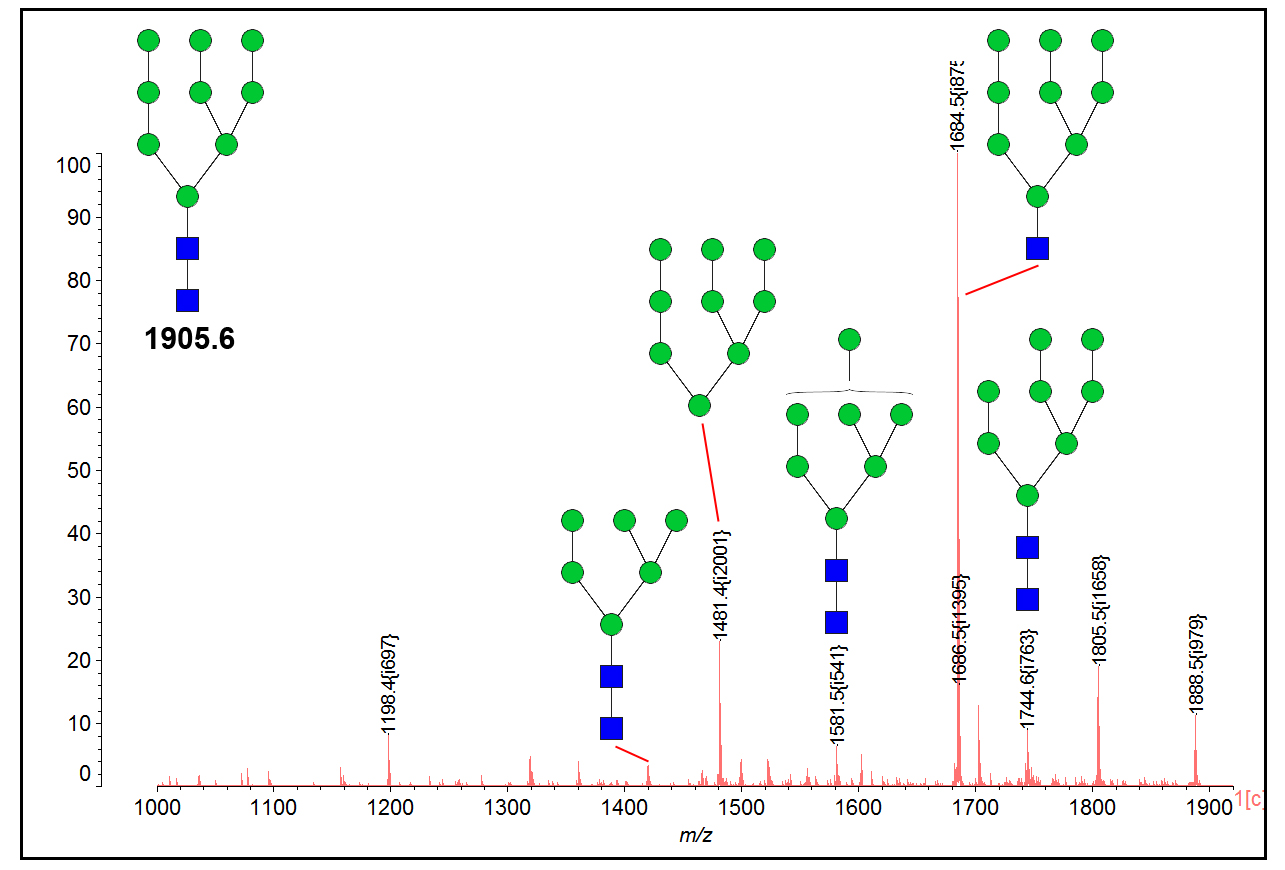

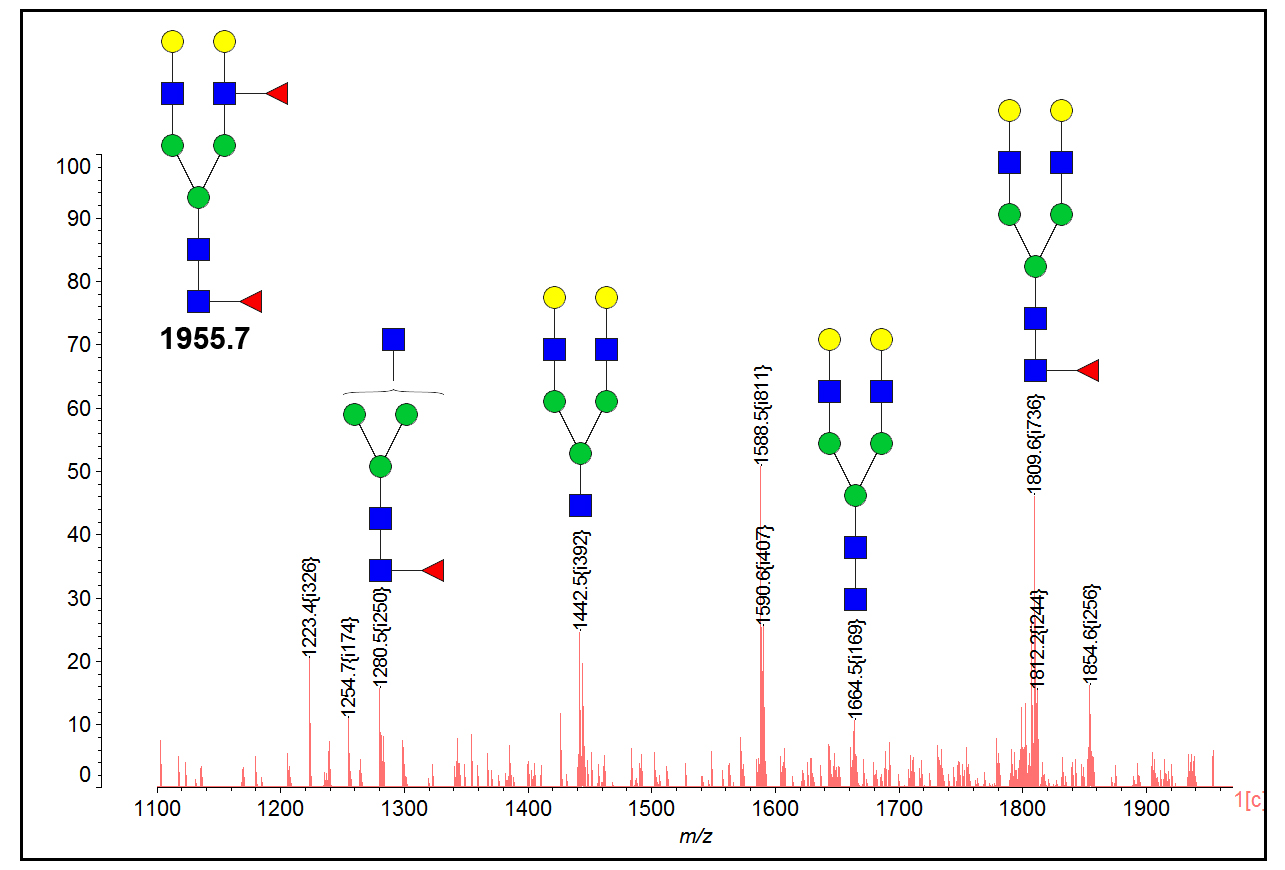

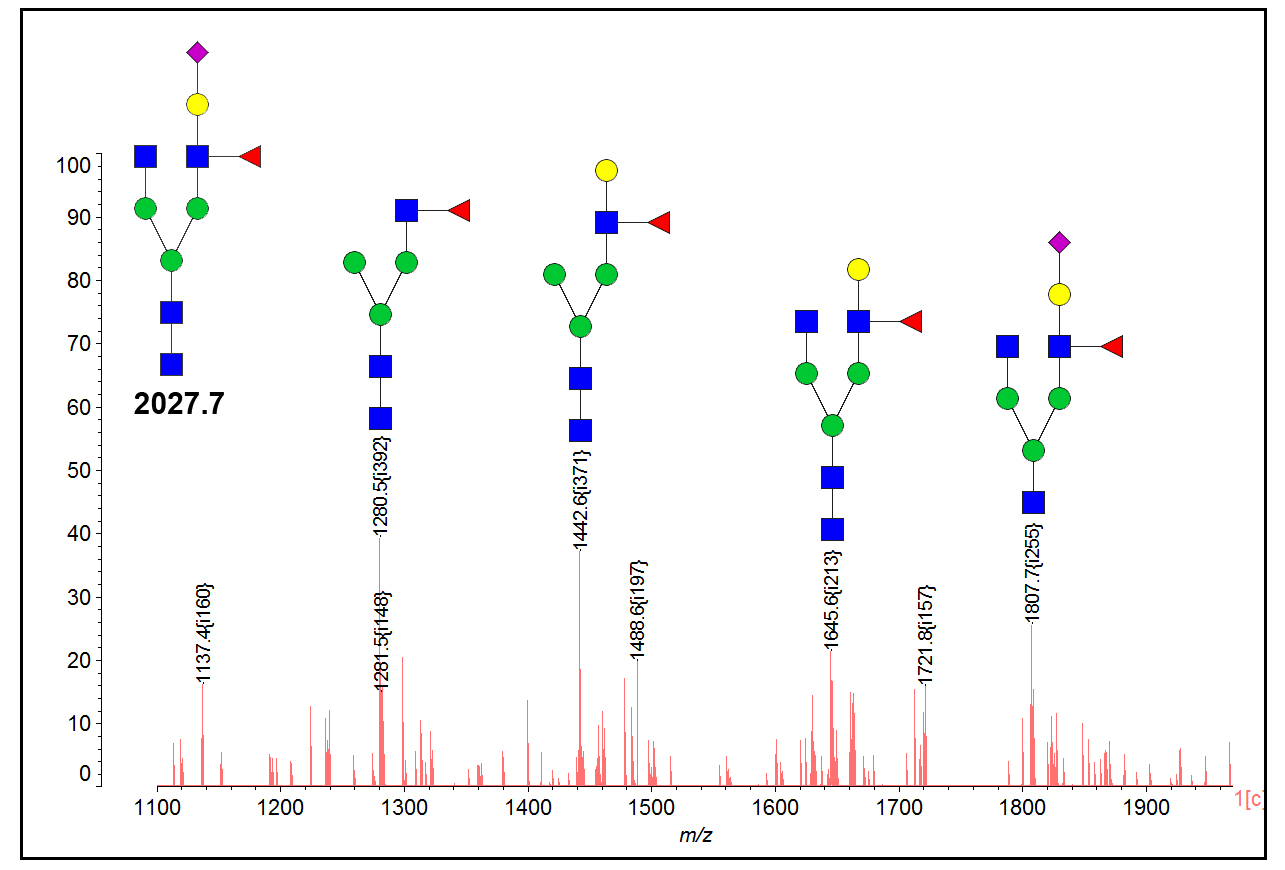

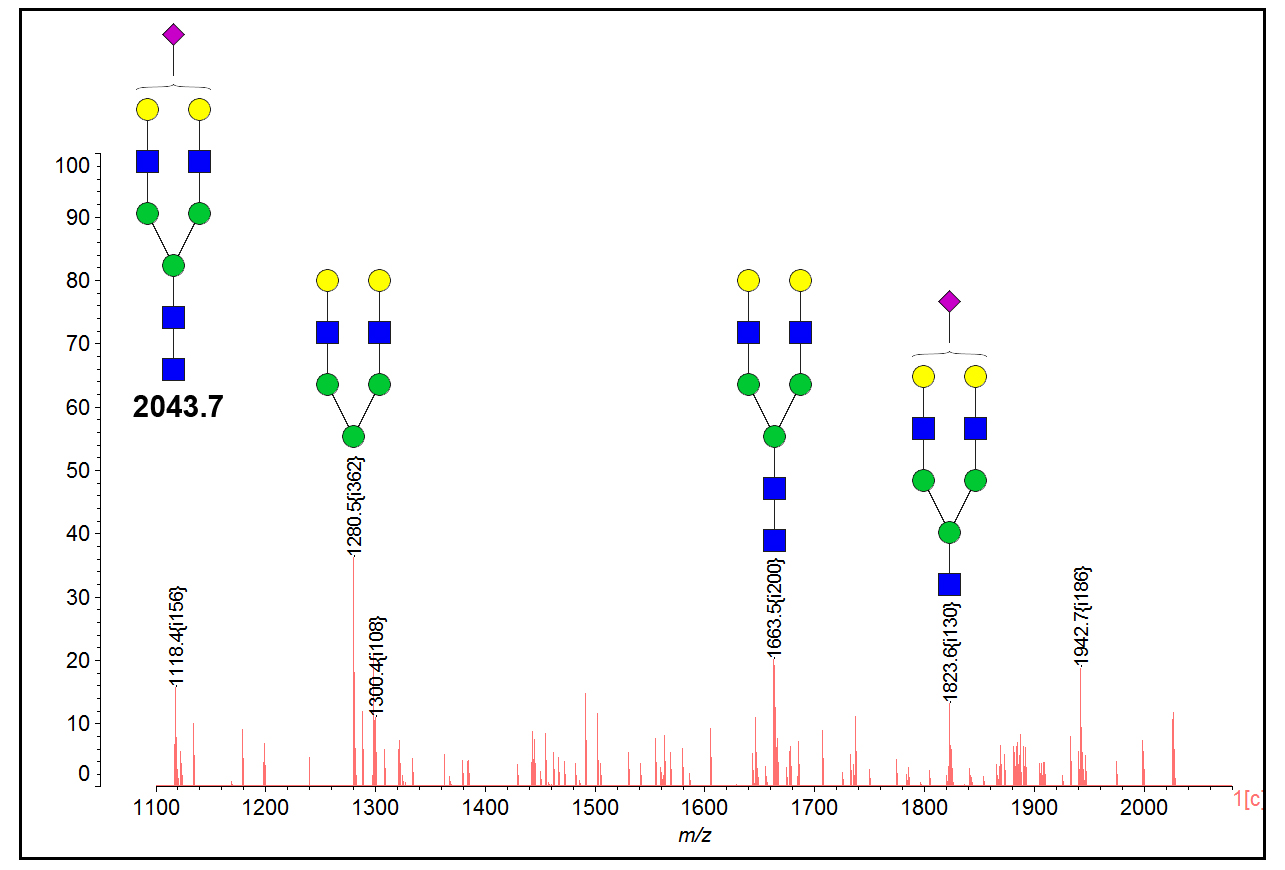

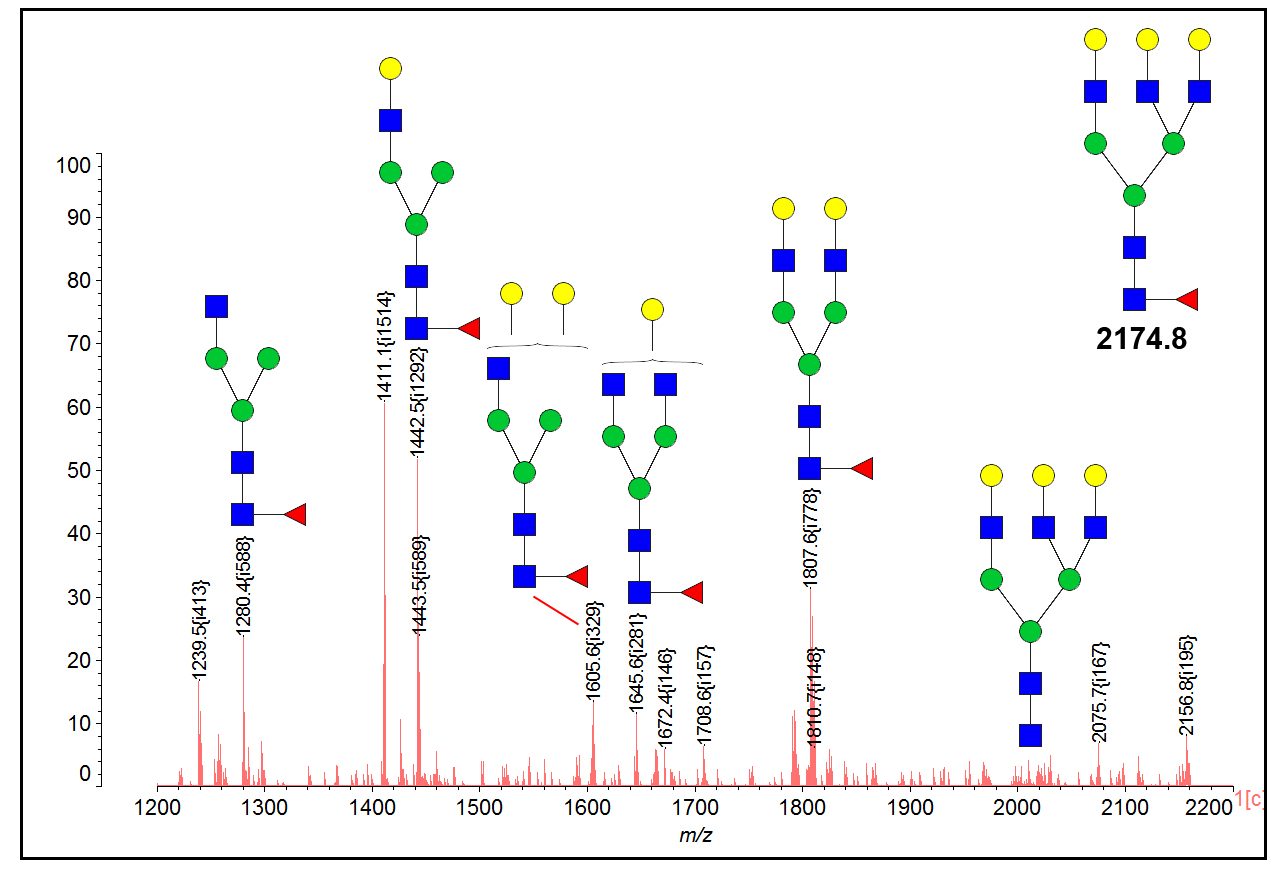

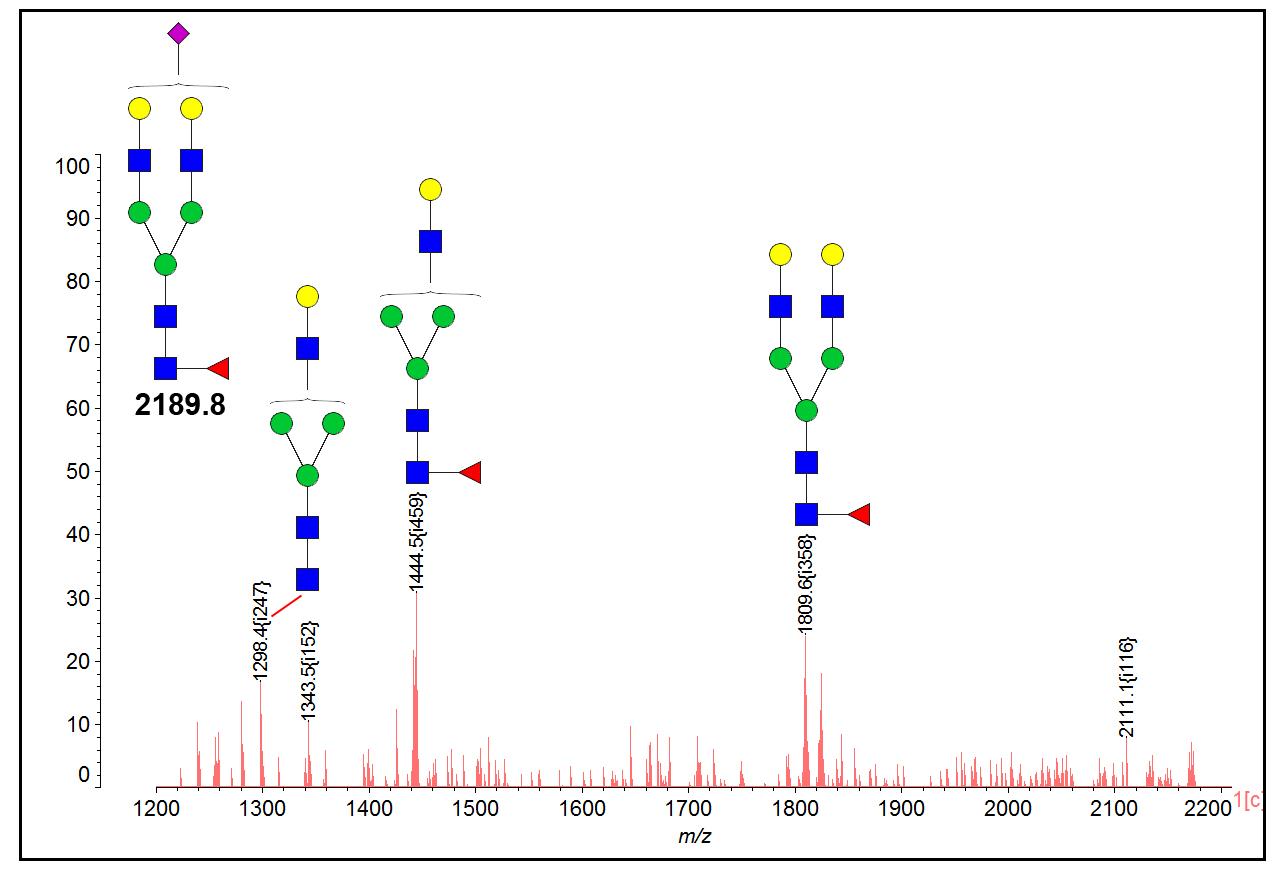

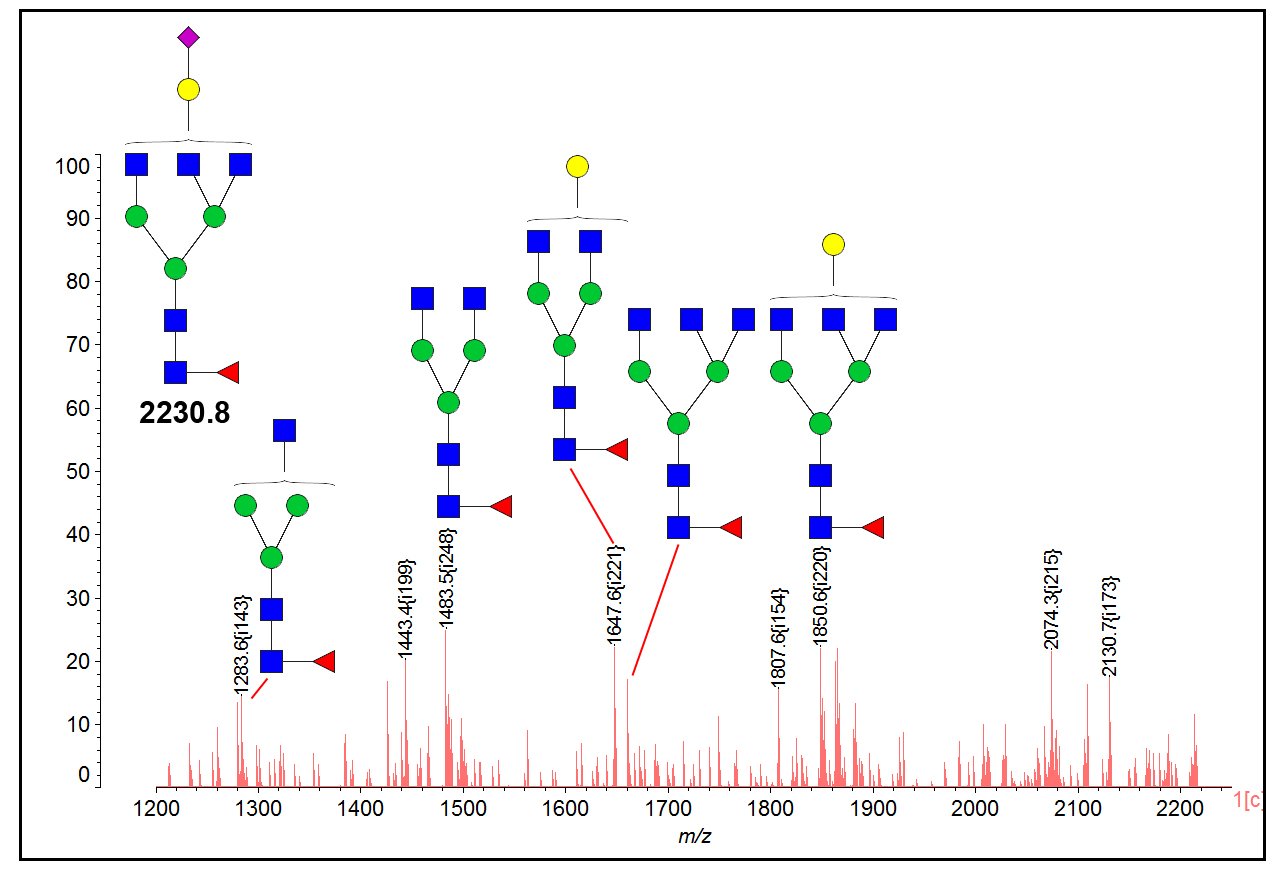

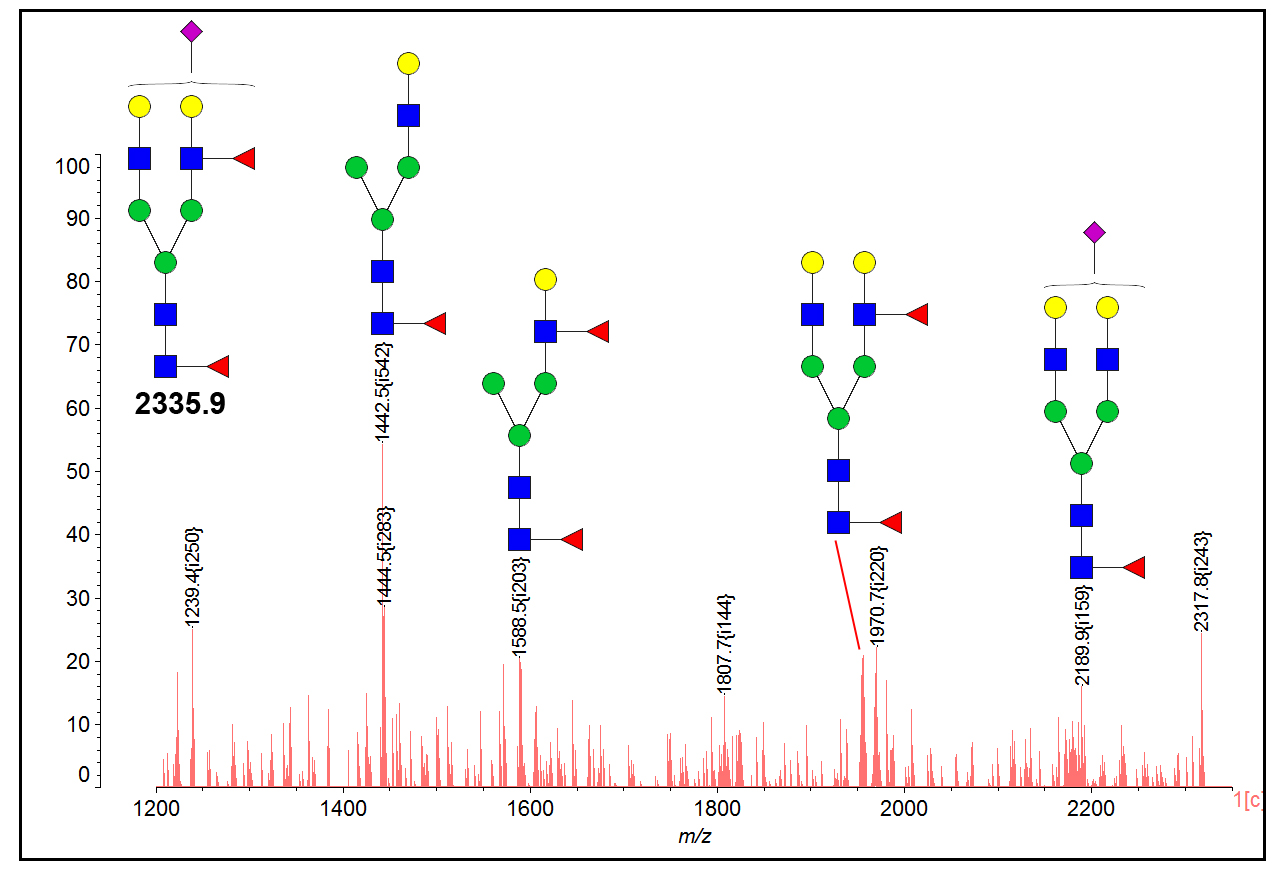

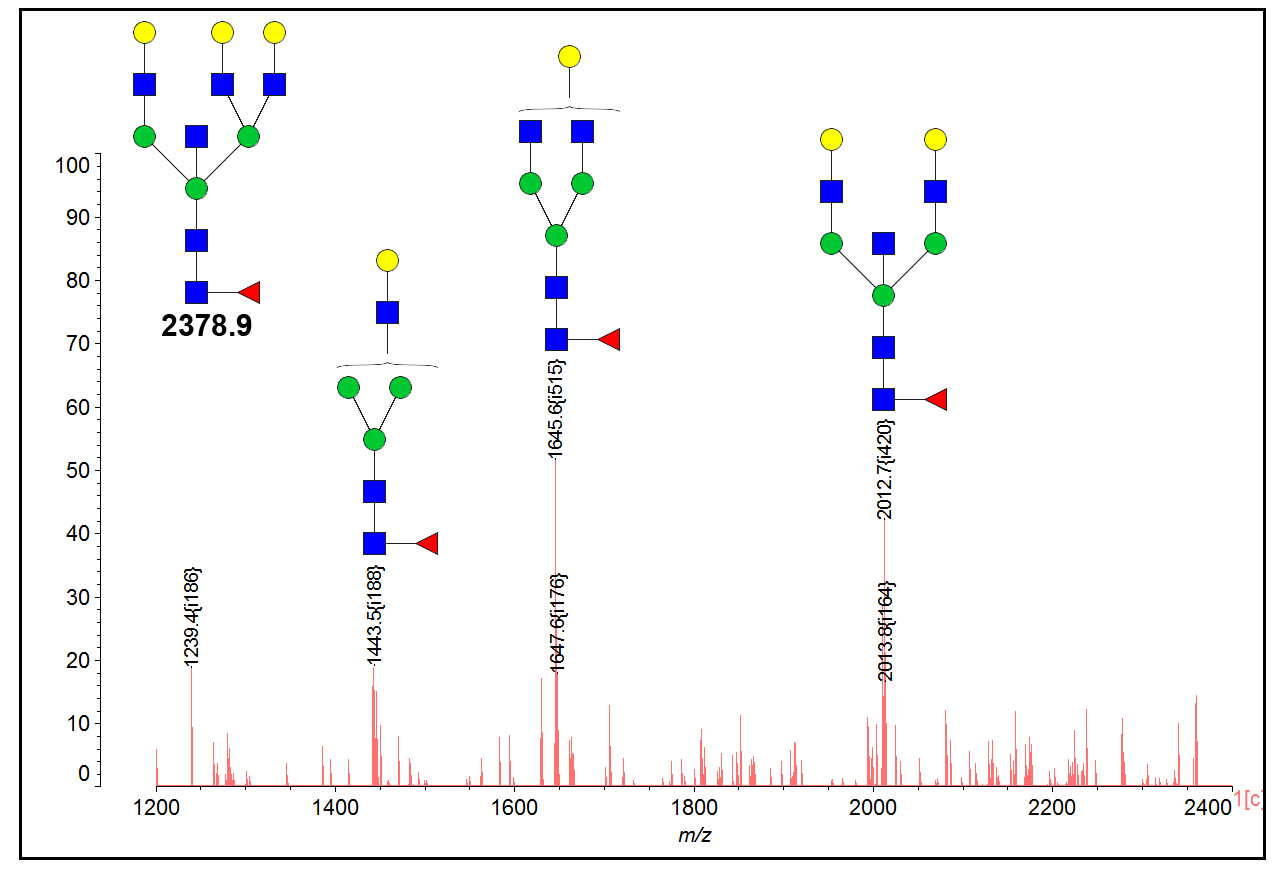

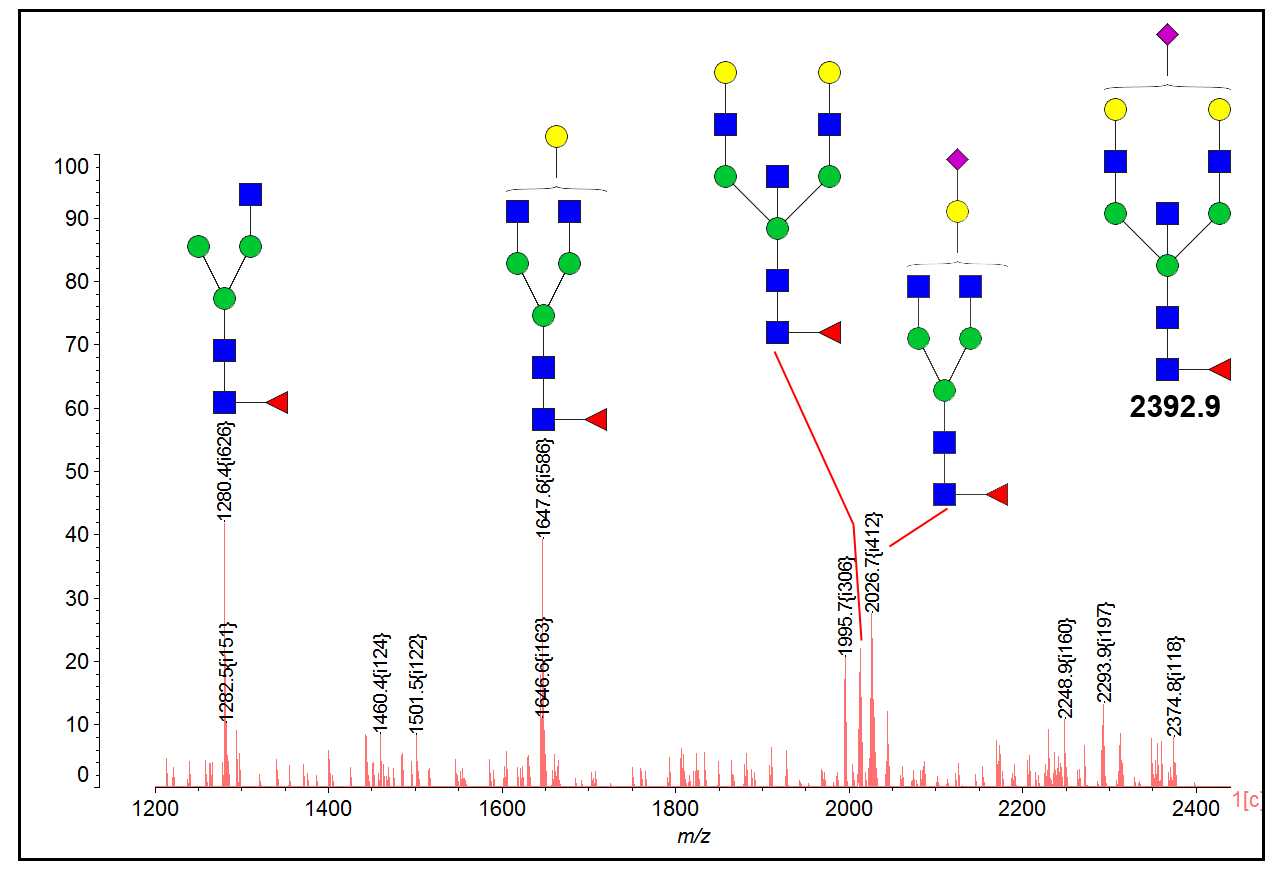

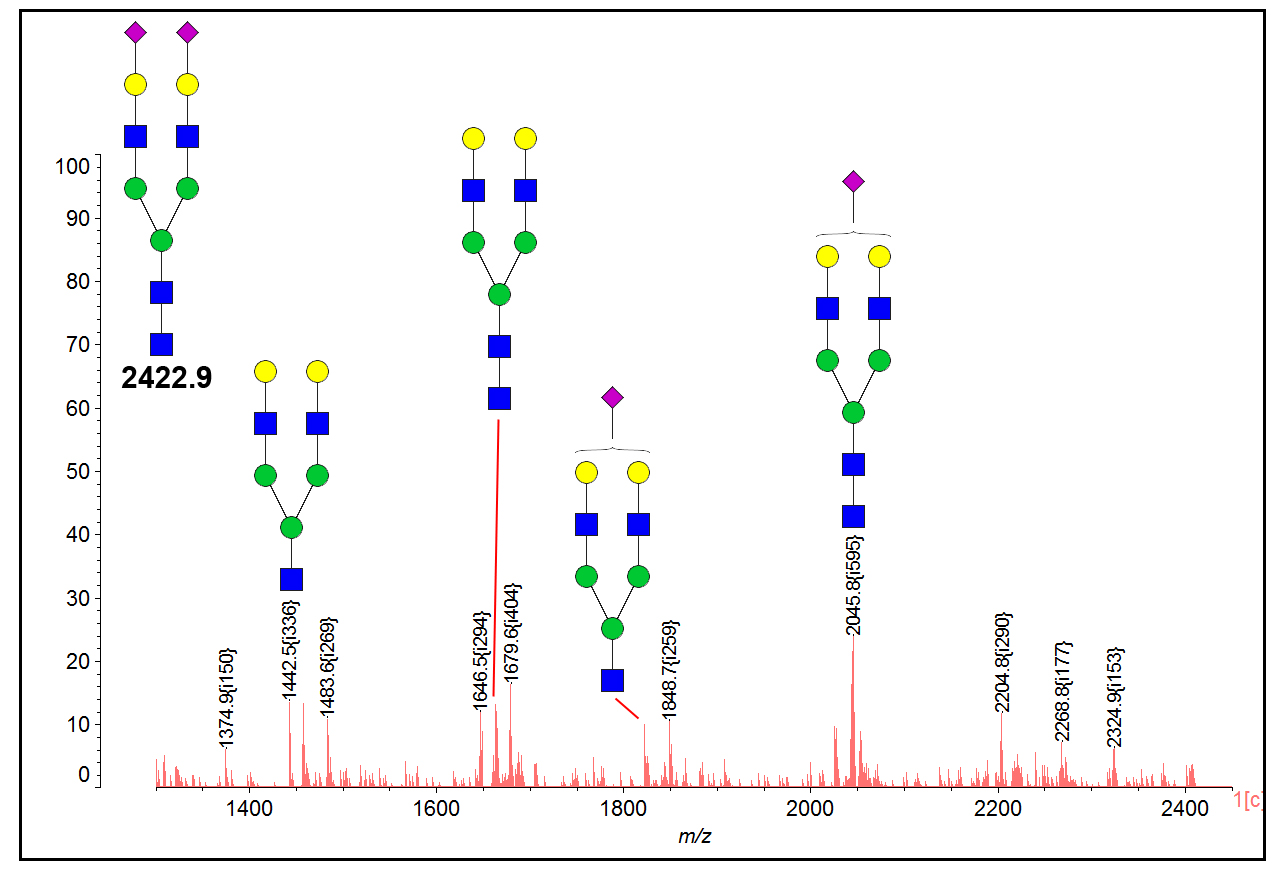

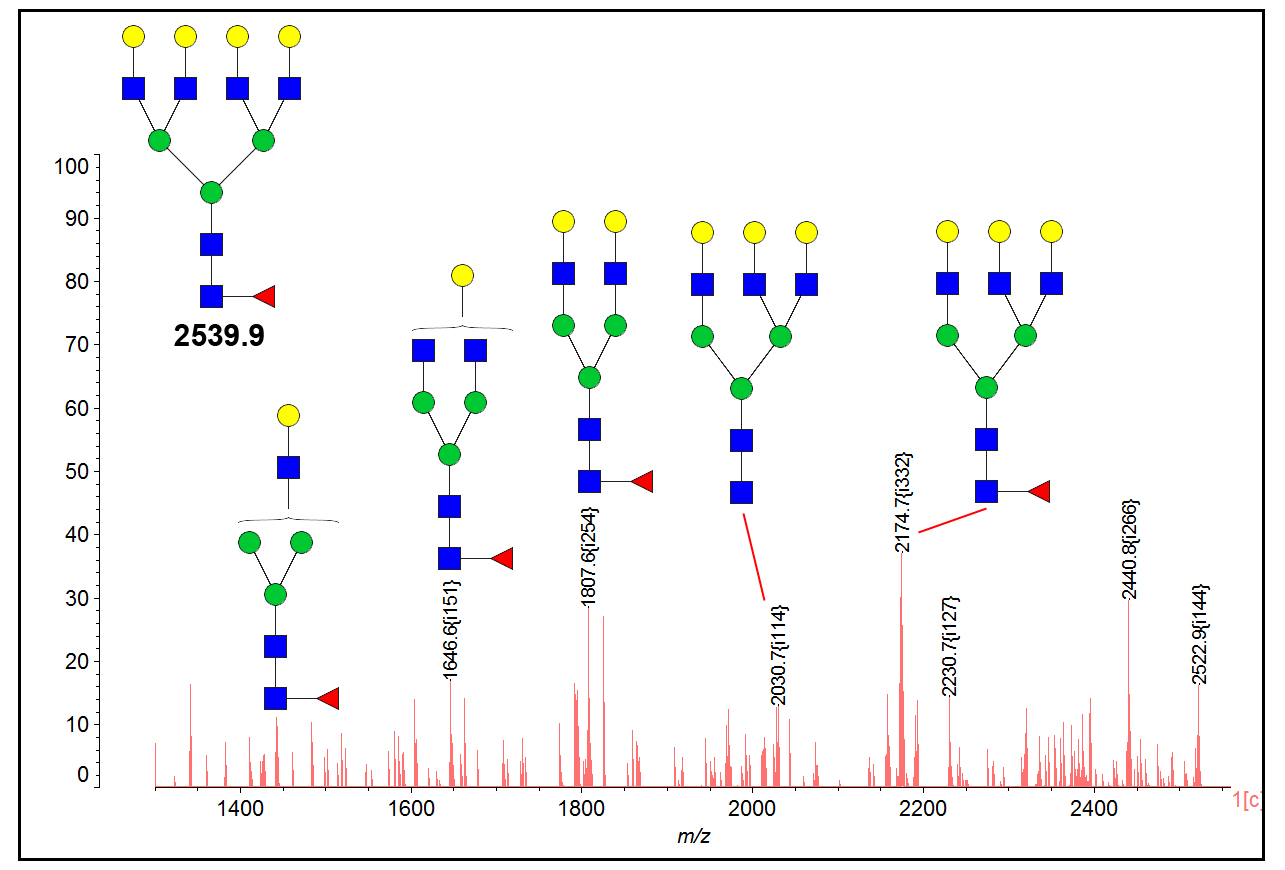

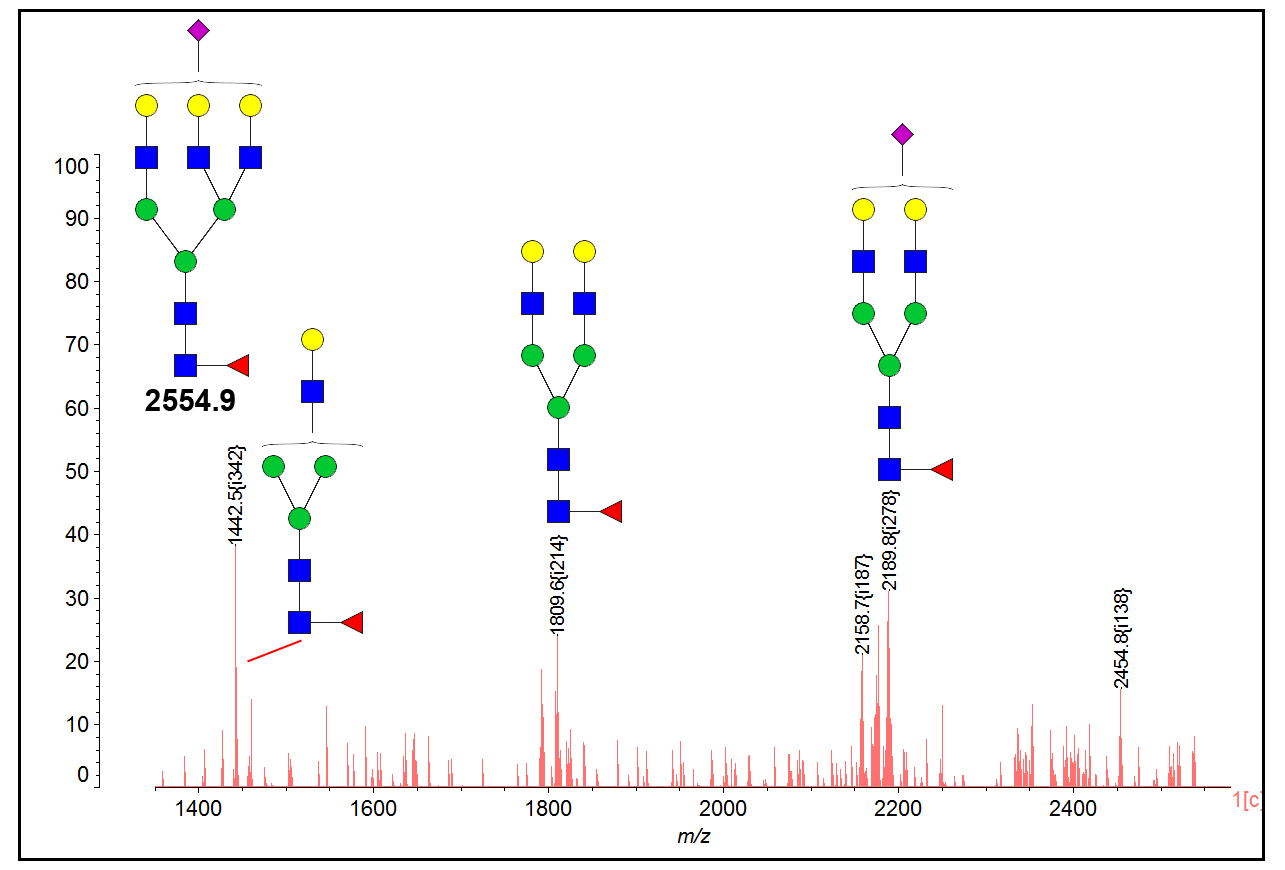

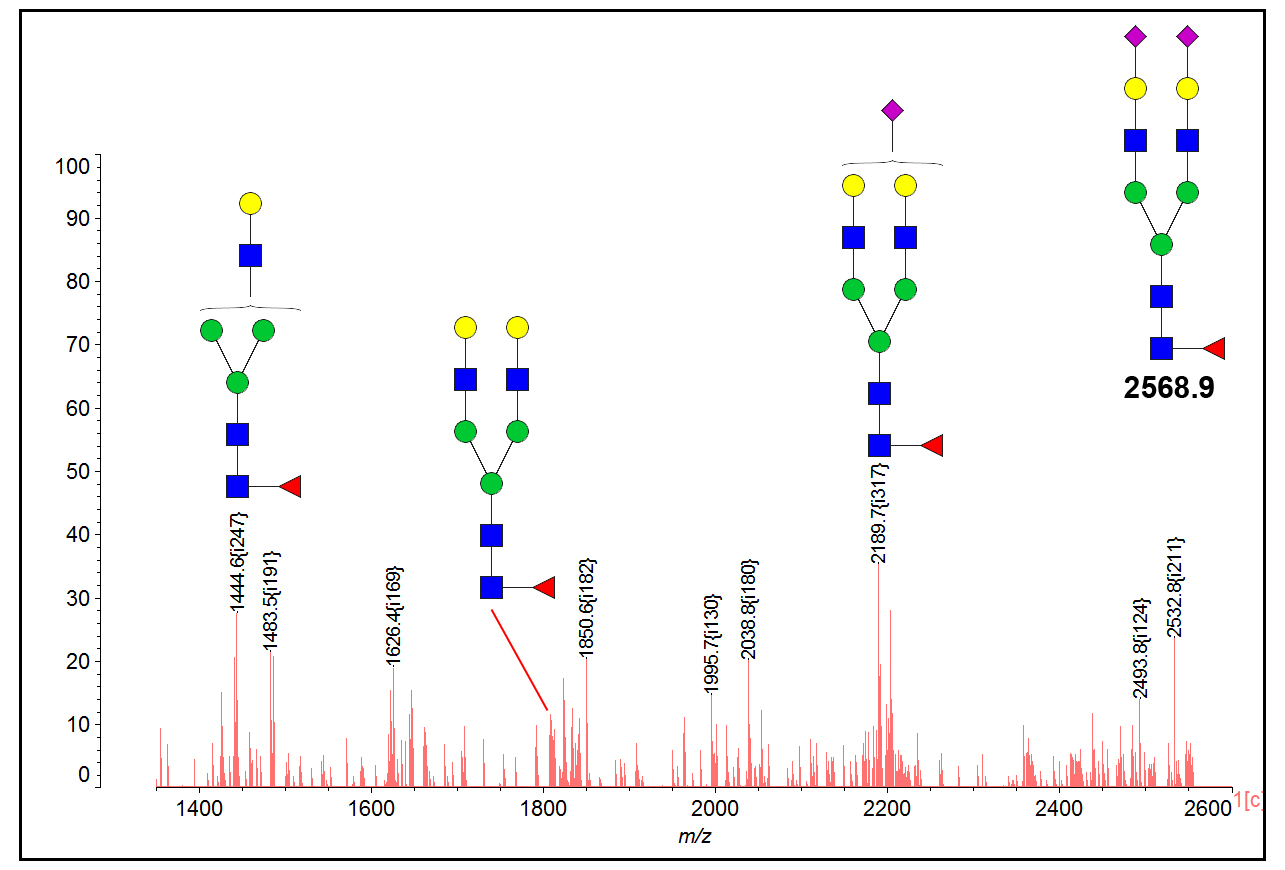

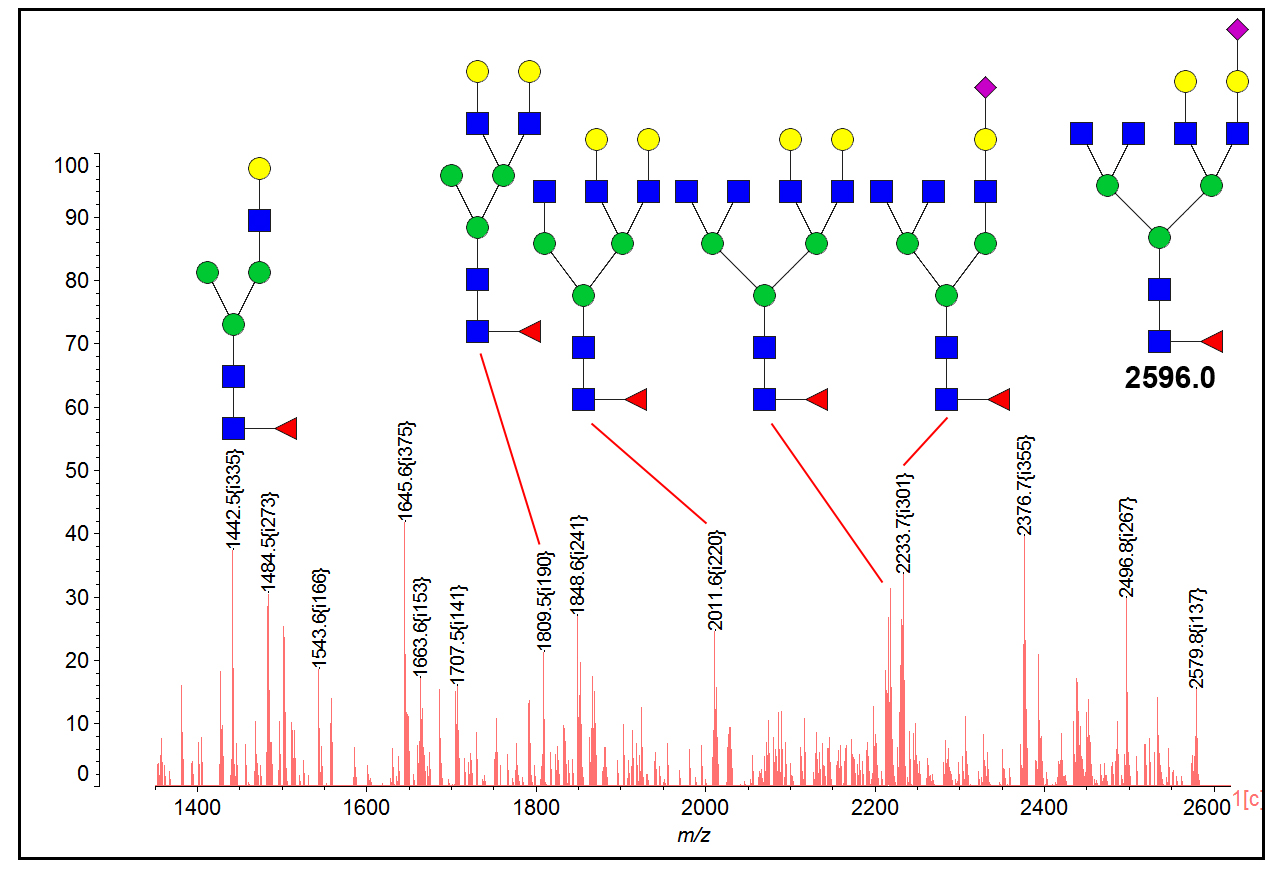

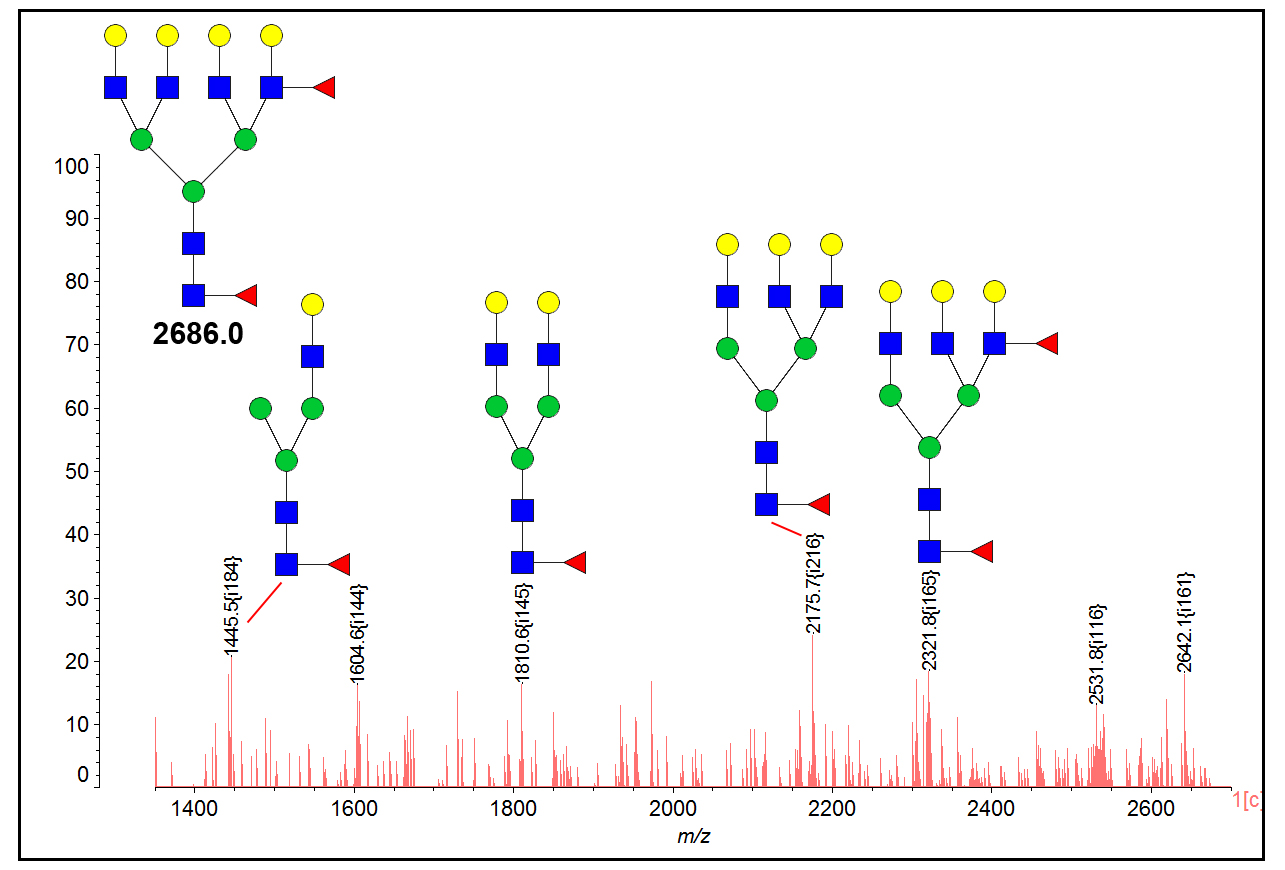

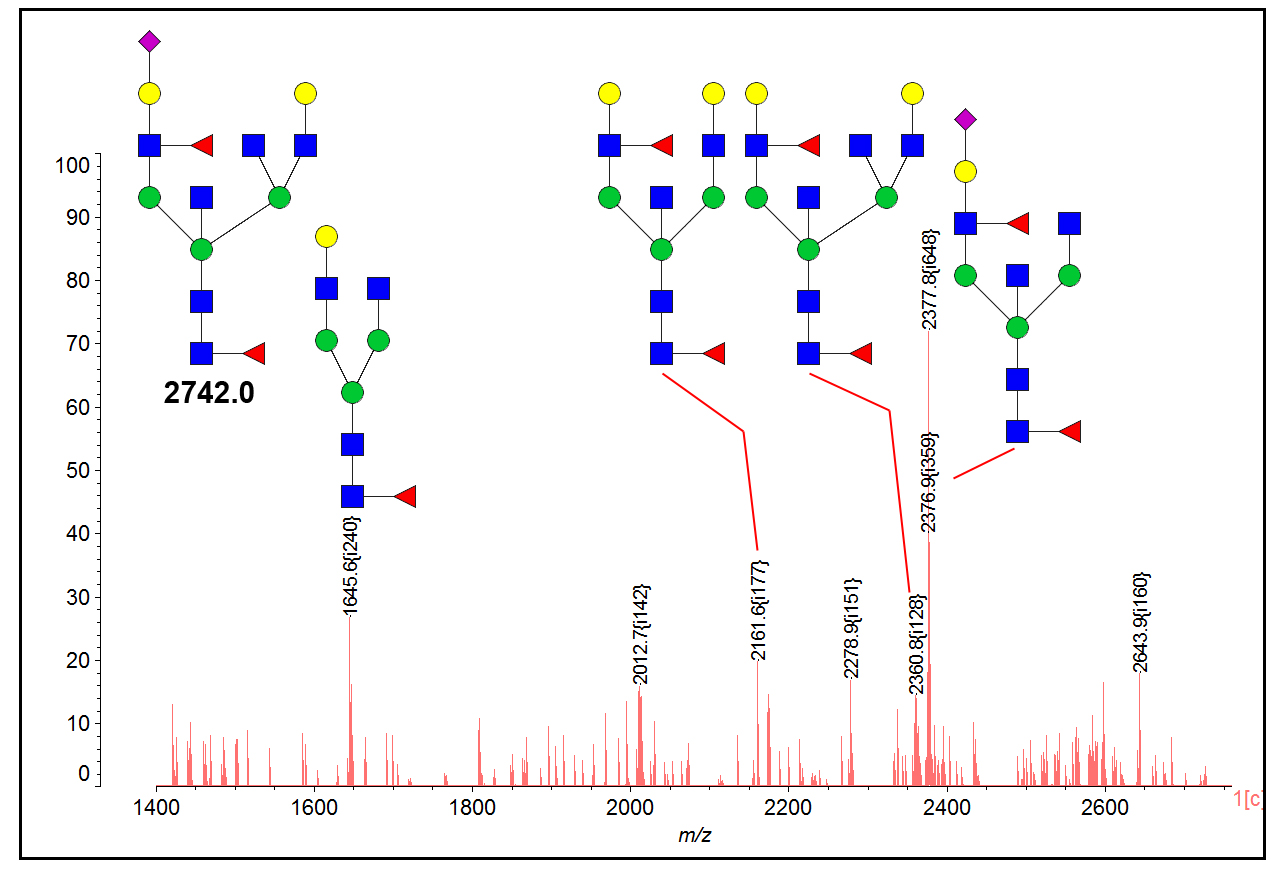

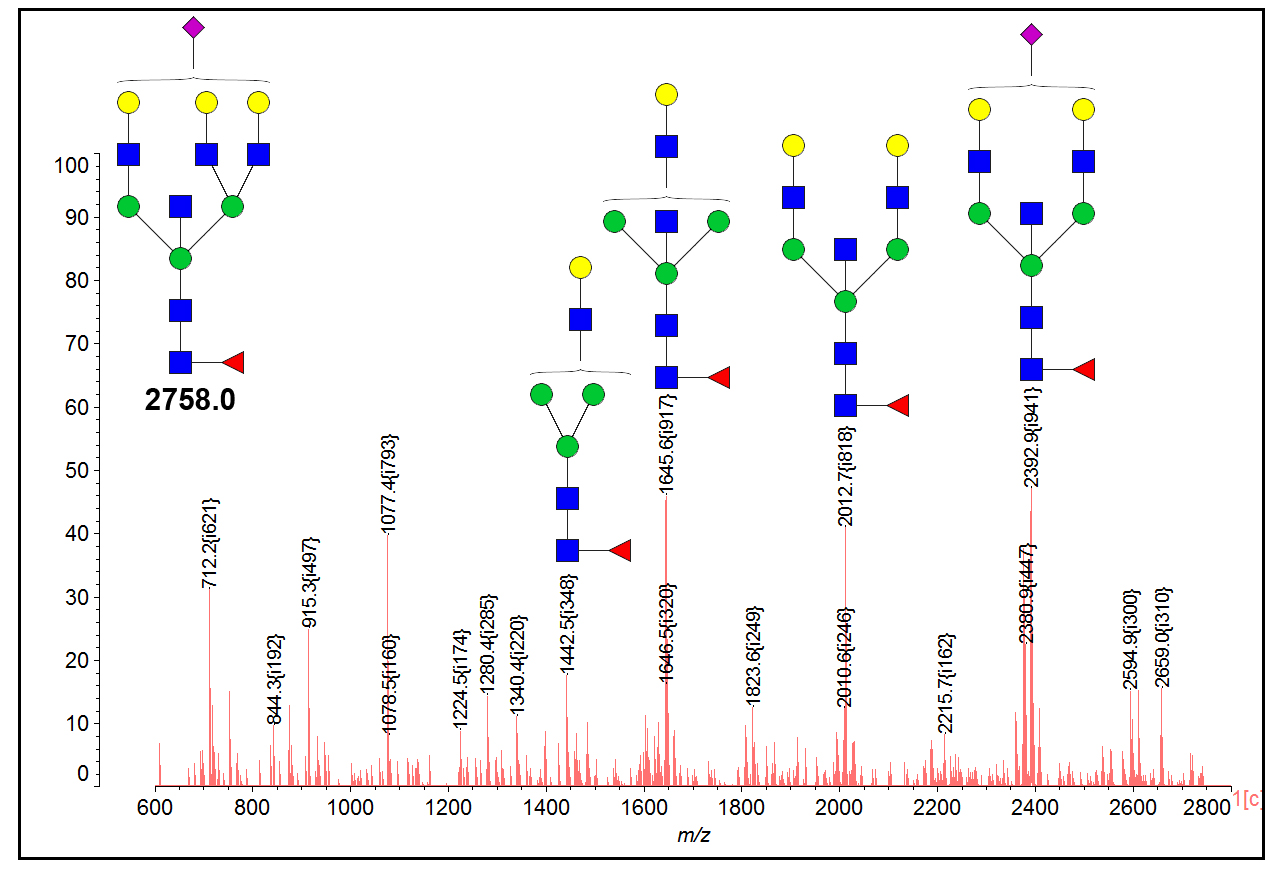

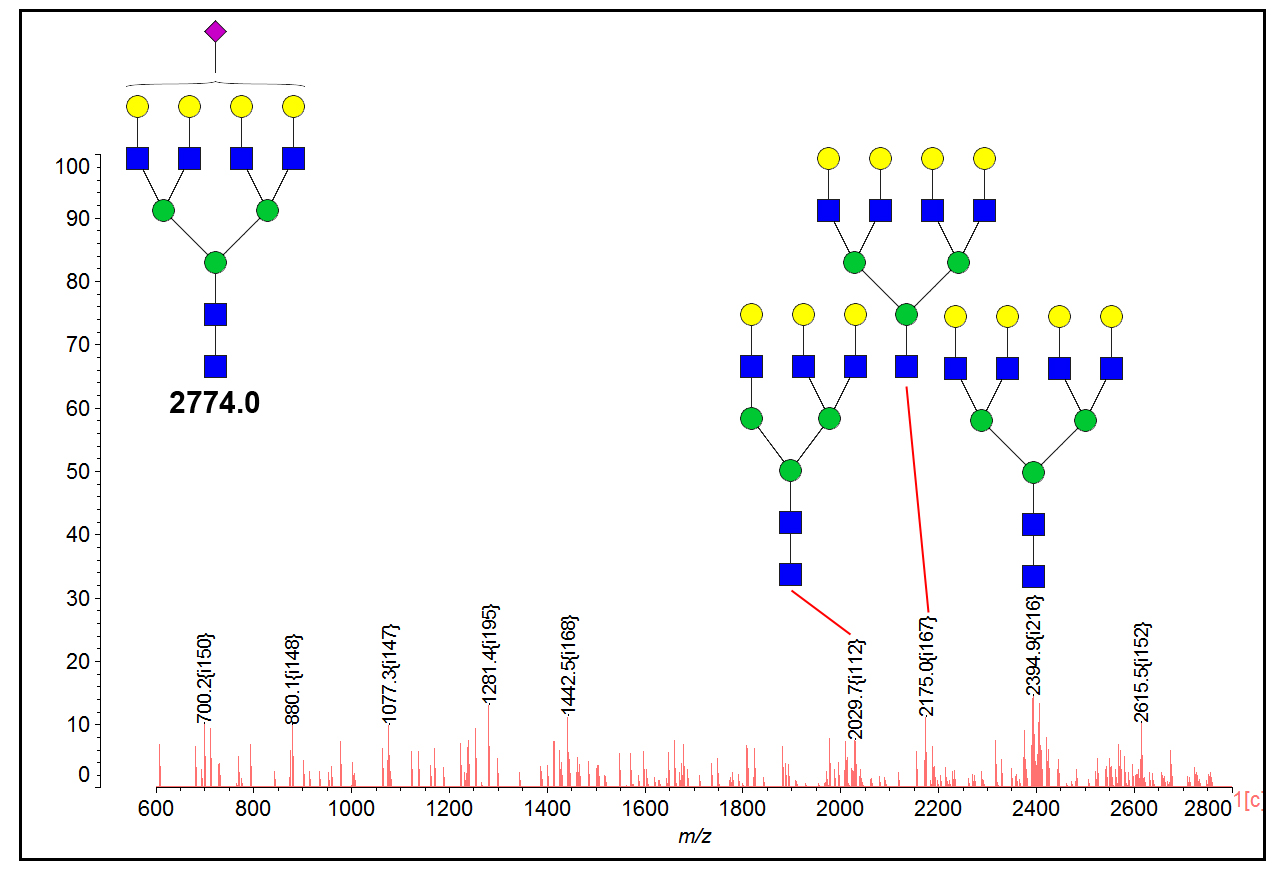

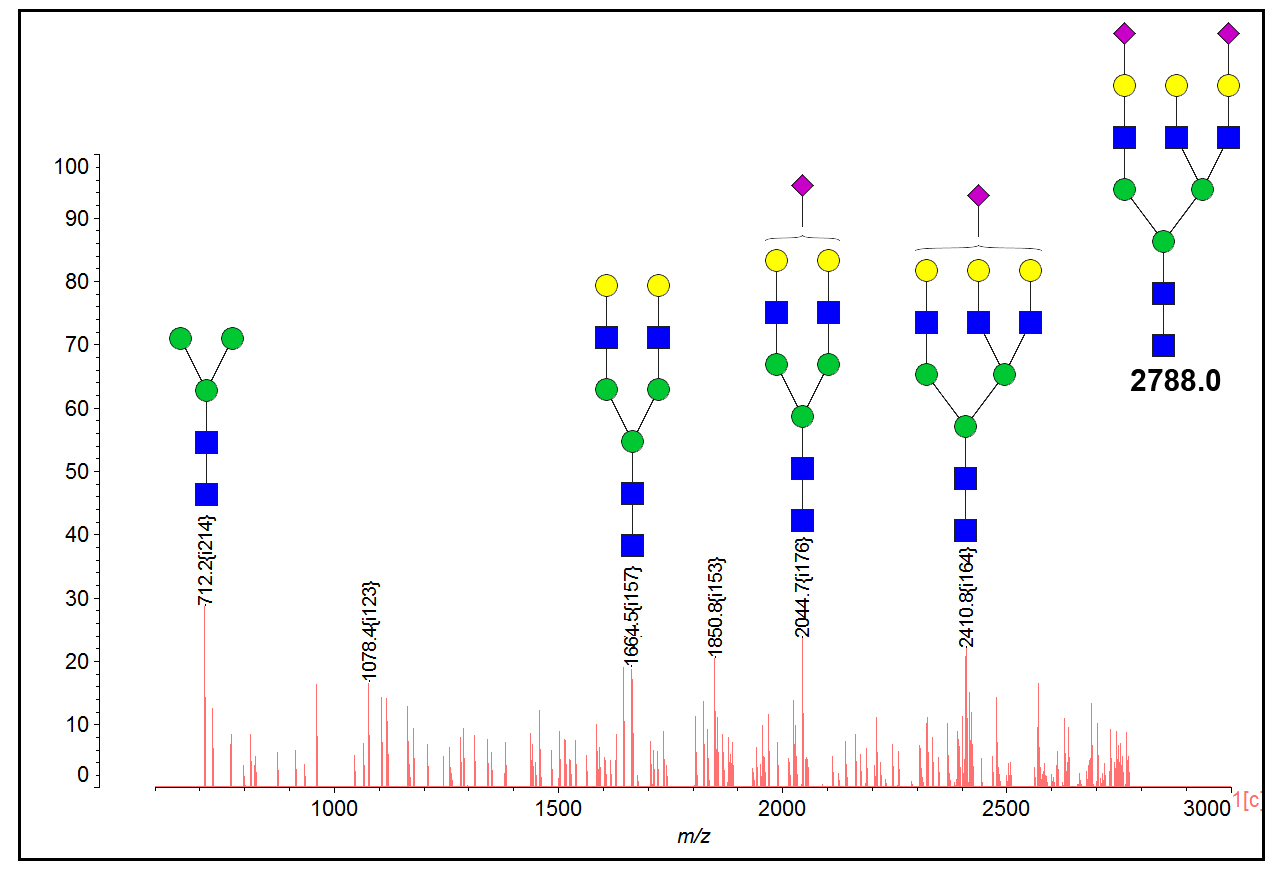

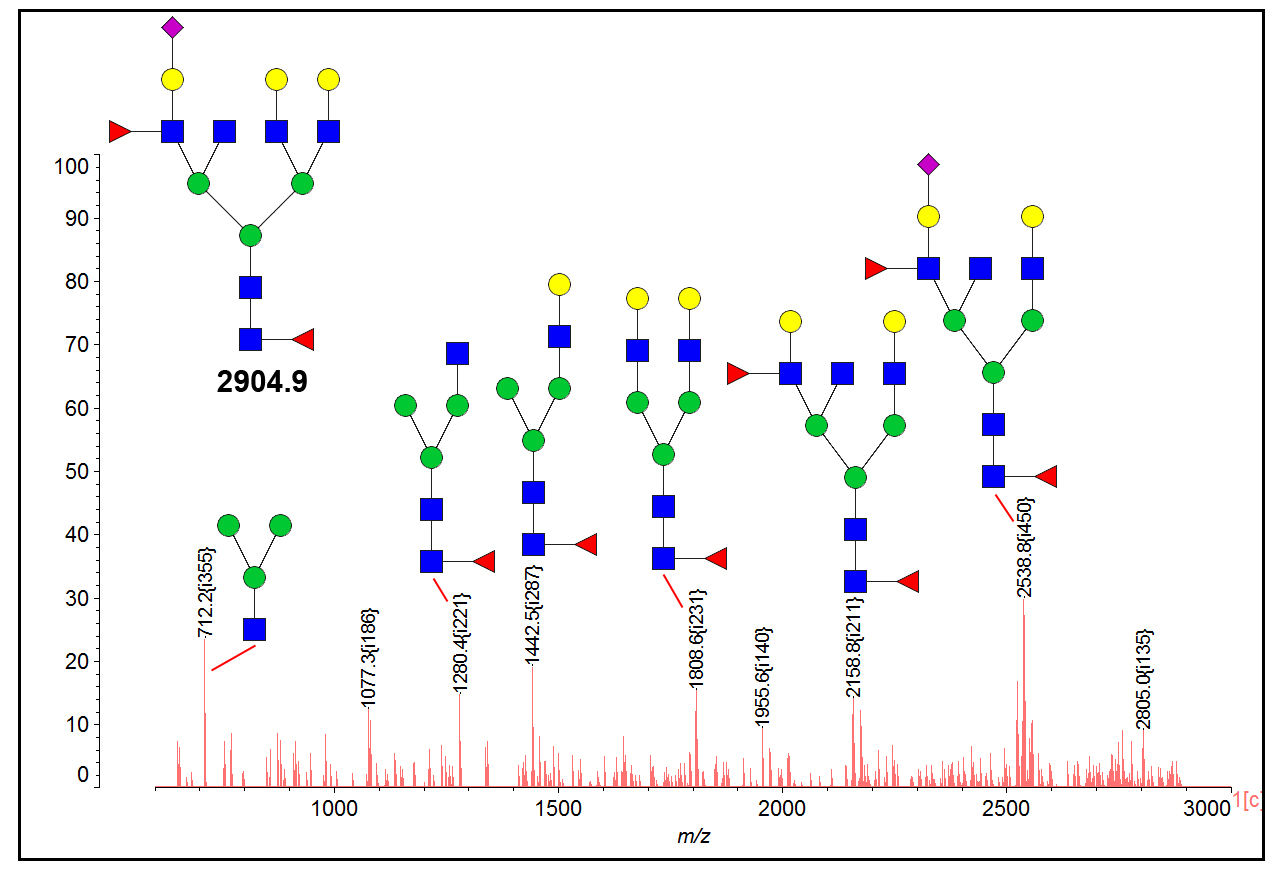

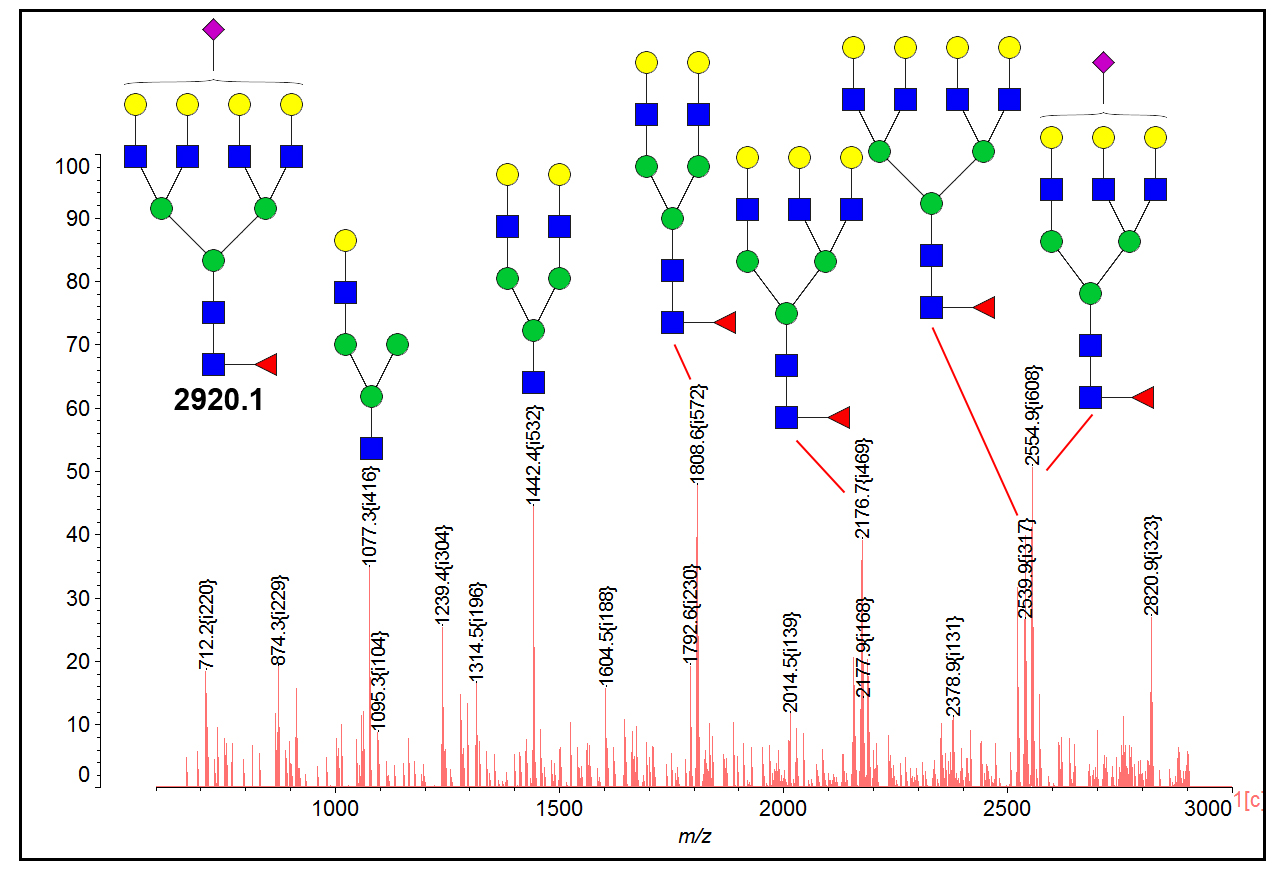

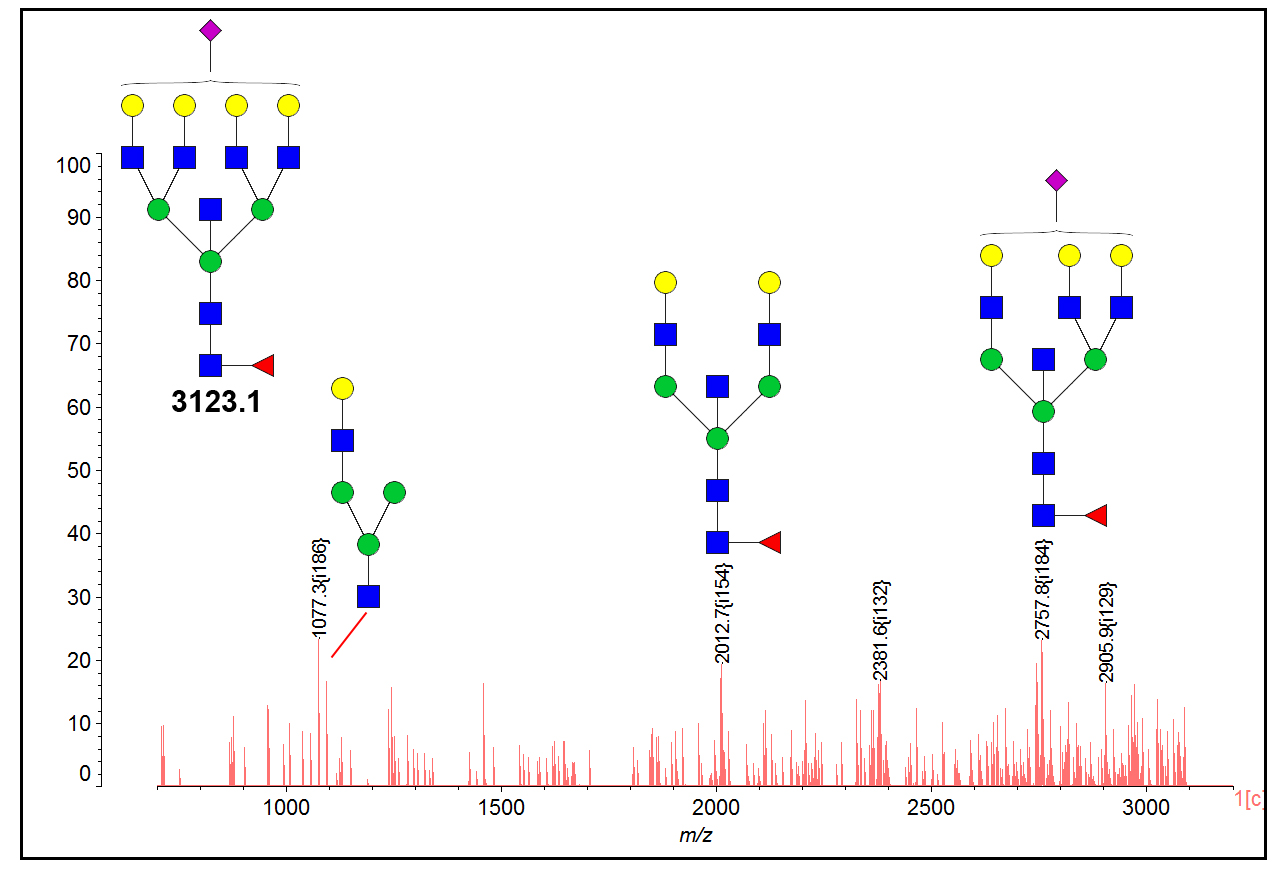

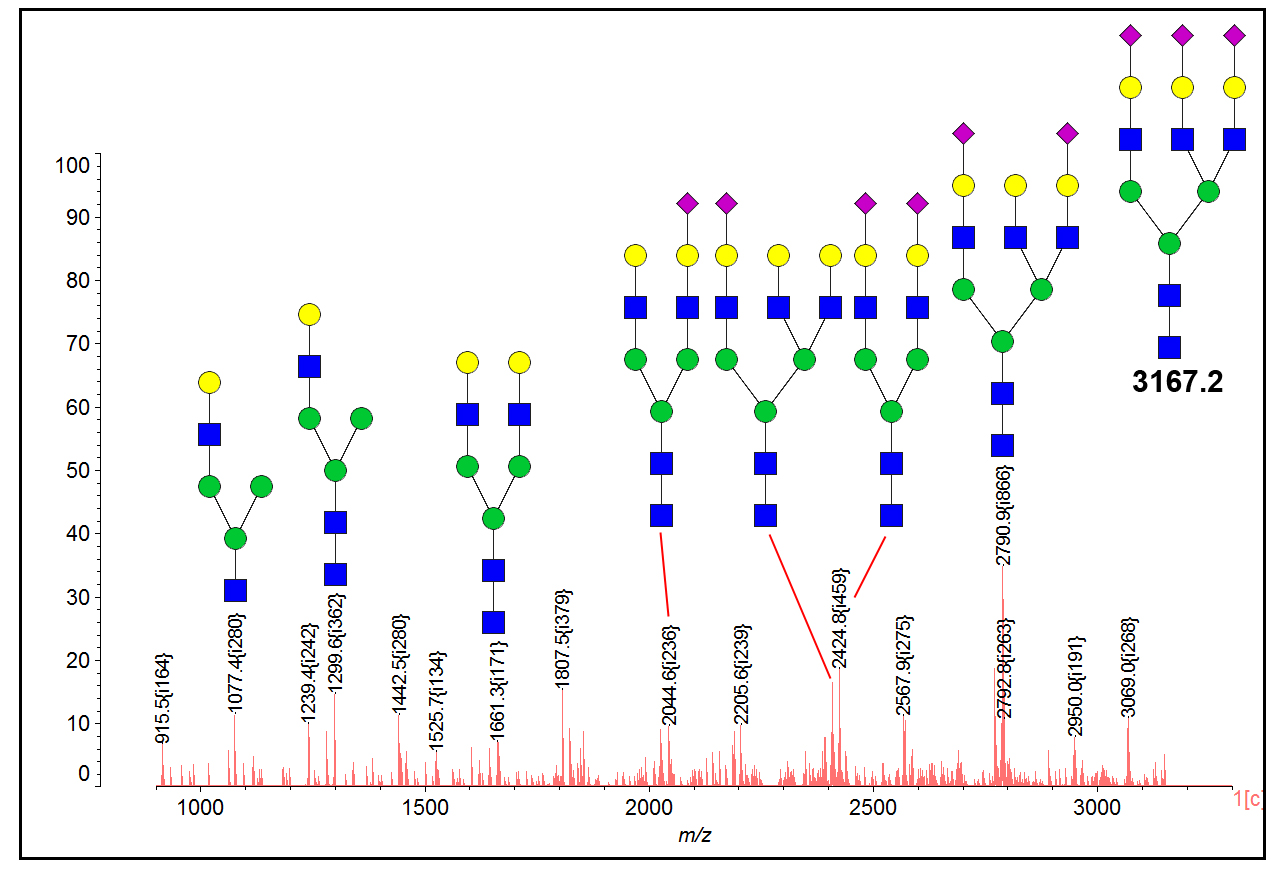

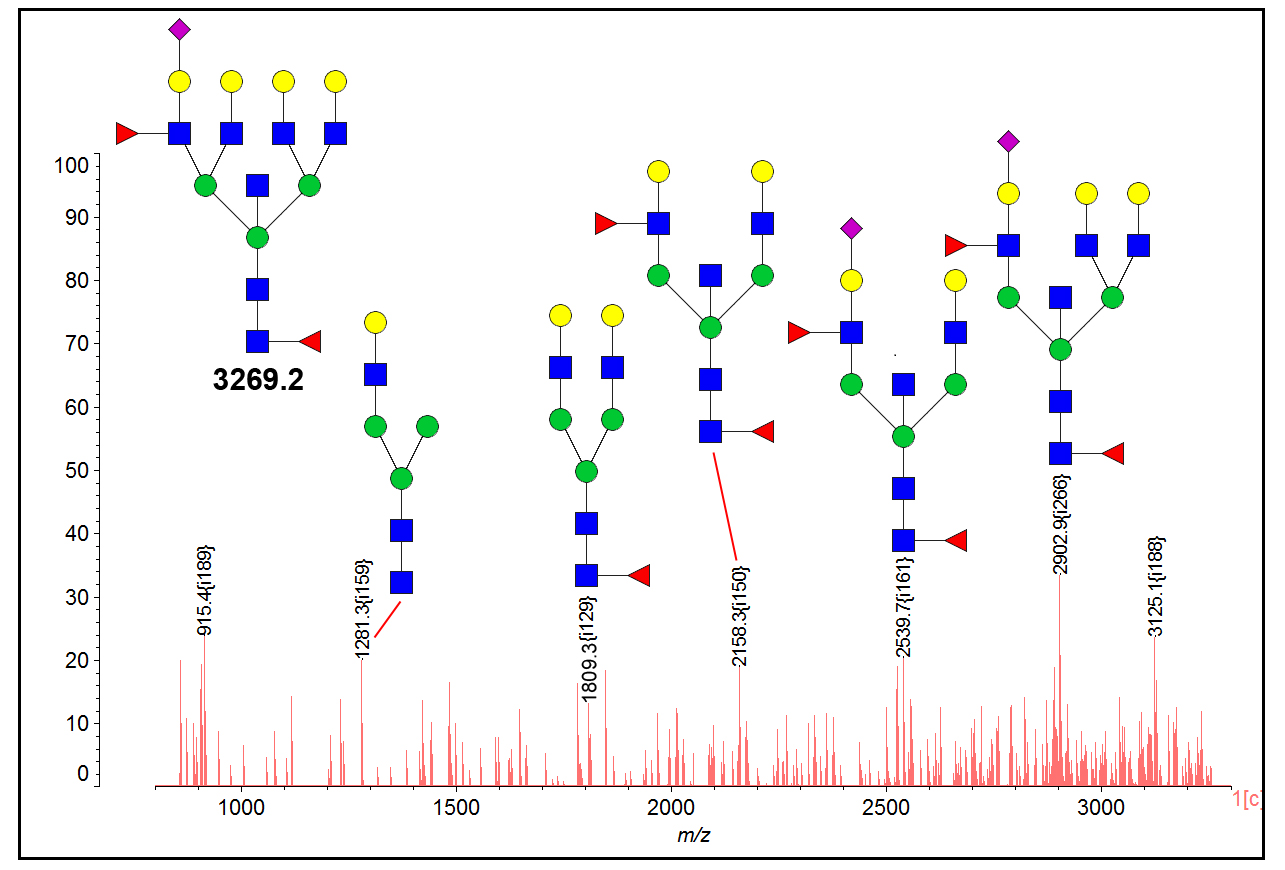

Supplement: Supplementary file 1 — Additional file 1: Figure S1. MALDI-MS identification of Oligomannoses. The internal standard (1 μL, 25 μM DP7) is spiked in the matrix for semi-quantification. Note: Man5 = F0N2H5S0. [file 12014_2017_9137_MOESM1_ESM.docx]
